# Honey and Cancer: Current Status and Future Directions

**DOI:** 10.3390/diseases4040030

**Published:** 2016-09-30

**Authors:** Laura M. Porcza, Claire Simms, Mridula Chopra

**Affiliations:** Institute of Biomedical and Biomolecular Science (IBBS), School of Pharmacy and Biomedical Sciences, University of Portsmouth, Hampshire, Portsmouth PO1 2DT, UK; Laura.Porcza@myport.ac.uk (L.M.P.); Claire.Simms@port.ac.uk (C.S.)

**Keywords:** honey, cancer, phenolic, flavonoid, proliferation, inflammation, apoptosis, angiogenesis, fibrobalsts, invasion

## Abstract

Cancer is a leading cause of death worldwide and poses a challenge to treatment. With overwhelming evidence of the role played by diet and lifestyle in cancer risk and prevention, there is a growing interest into the search for chemopreventative or chemotherapeutic agents derived from natural products. Honey is an important source of bioactive compounds derived from plants and recent years have seen an increased interest in its anticancer properties. This review examines the role of honey in targeting key hallmarks of carcinogenesis, including uncontrolled proliferation, apoptosis evasion, angiogenesis, growth factor signalling, invasion, and inflammation. The evidence for honey as an adjunct to conventional cancer therapy is also presented. The review also highlights gaps in the current understanding and concludes that, before translation of evidence from cell culture and animal studies into the clinical setting, further studies are warranted to examine the effects of honey at a molecular level, as well as on cells in the tumour environment.

## 1. Introduction

Cancer is one of the most common causes of death and represents a significant health burden [1]. In spite of considerable research input, this pervasive condition remains a challenge to prevent and treat. Conventional methods to treat cancer have severe side effects necessitating the search for novel, less toxic therapies. Approximately 90%–95% of cancer cases are thought to be related to the environment and lifestyle of an individual [2], highlighting the potential role of diet in carcinogenesis [3]. In recent years there has been increased interest in the search for chemopreventive and chemotherapeutic agents derived from food or natural products. The relative safety of food-derived compounds makes this an attractive alternative compared to conventional cancer therapy.

Natural honey is produced by bees and contains over 200 compounds, consisting mainly of sugars (75% monosaccharides: glucose and fructose; 10%–15% disaccharides: sucrose, maltose, etc.) and water, as well as enzymes, vitamins (Vitamin B6, riboflavin, niacin, thiamine, etc.), minerals, phenolic compounds (flavonoids, phenolic acids), volatile compounds, and pigments [4,5,6,7]. Honey has been used for centuries as a food source and medicine, and current research suggests it may be a beneficial aid to cancer therapy. Reactive oxygen species (ROS) and inflammation play an important role in the process of carcinogenesis [8,9]. The antioxidant and anti-inflammatory action of honey is well documented [10,11,12,13,14] and related to its phenolic constituents [4,5,15,16]. These include phenolic acids such as caffeic acid, ellagic acid, gallic acid, syringic acid, chlorogenic acid, p-coumaric acid, ferulic acid, and the flavonoids chrysin, kaempferol, catechin, quercetin, galangin, luteolin, pinocembrin, pinobankskin, and myricetin [17,18]. Caffeic acid, benzoic acid, and gallic acid are the most common phenolic acids, and flavonoids such as quercetin, catechin, kaempferol, luteolin, and apigenin are shared among many honey types. The highest content is often found in manuka honey, a monofloral honey derived from new Zealand, tualang honey, a Malaysian multifloral honey and the monofloral buckwheat honey obtained from several geographical sources, while the lowest phenolic content is seen in two monofloral varieties: gelam honey (*Melaleuca* sp.) and acacia honey (*Pseudoacacia*) [7,17,19,20,21]. The phenolic content of honey varies between 86 and 1141 mg/kg and is related to several factors including their floral source, geographical origin [7,17,19,20,21], as well as the HPLC method used for analysis in the laboratory [22,23,24,25]. It is also worth noting that many researchers tend to report the total phenolic content of honey in gallic acid equivalents and the Folin–Ciocalteau method used for measuring the total phenolics tends to overestimate the values [20]. Overall, honey contains constituents that may confer its anticancer properties, but effectiveness is likely to vary with its composition. There are limited studies on the bioavailability of phenolics from honey, however supplementation with 1.5 g/kg buckwheat honey (containing ~1.171 mg of phenolic antioxidants per gram) has been reported to produce significant increases in plasma phenolics 2 h after supplementation, the levels remaining high for up to 6 h [26].

There are a few reviews that have examined the anticancer properties of honey. Among the ones published within the last two years, a review by Jagnathan et al. [27] focused only on the antiproliferative and apoptotic potential of honey. A second review from the same laboratory discussed, in depth, the anticancer potential of honey and of its constituents against colon cancer only [28]. Another review by Erejuwa et al. [29] thoroughly covered the anticancer potential of honey against several cancer types and also described several possible mechanisms of action. However this review does not mention the concentrations of honey nor the percentage inhibitory effect. Furthermore, the effect of honey on the invasive properties of cancer cells, as well as the potential of honey being used as an adjunct to chemotherapy was not addressed either. The present review contains more detailed information which should complement the previous reviews. It also provides a critical appraisal of the published data, highlights gaps in the current understanding, and offers suggestions for further studies, which are warranted before recommendations for clinical studies can be made.

## 2. Honey and Cancer

The development of cancer is a complex process beginning with alteration of the genetic material of healthy cells which, if not repaired, can result in abnormal proliferation, failure of apoptosis, and the development of tumours [30]. Multiple events collude to produce cancer, including activation of oncogenes, inactivation of tumour suppressor genes, deregulation of cell signalling pathways, growth factors and hormones, as well as many other interconnected complex processes [31]. The underlying commonality of cancer has been highlighted by Hanahan and Weinberg, who described hallmarks leading to the progression of a normal cell to a neoplastic state. These include replicative immortality and sustained proliferative signalling, resisting cell death/apoptosis, angiogenesis induction, evading growth suppressors, and activating invasion and metastasis [31]. The heterogeneity in cellular genotypes and phenotypes and multiple mechanisms that underlie the pathology makes cancer a difficult disease to treat. Targeting any one specific mechanism, as seen in many conventional therapies, often does not achieve the desired outcome.

**Figure 1:**
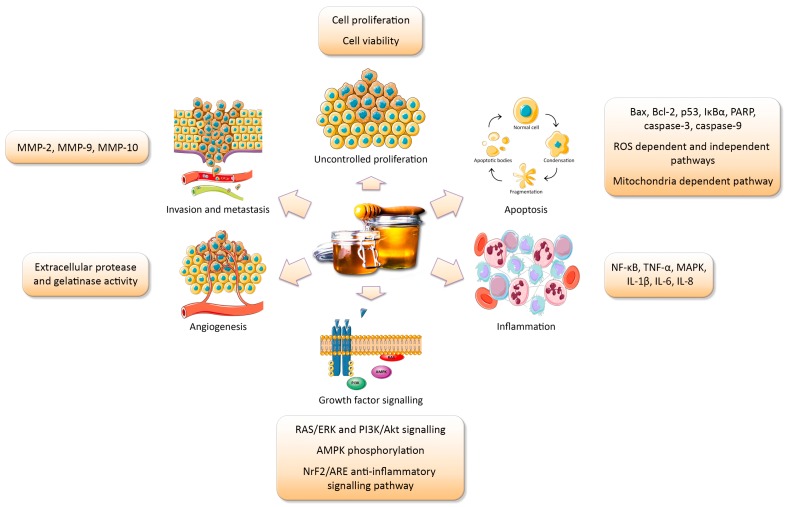
The carcinogenic processes likely to be targeted by honey.

The effect of honey treatment on these key hallmarks of cancer, as well as on inflammatory signalling, will be discussed in this review (Figure 1), with reference to the dosage, composition, and different types of honey examined.

### 2.1. The Antiproliferative Properties of Honey

Aberrant proliferation is a defining feature of tumour cells and is a key target for both conventional chemotherapeutics and novel therapies. Cell cycle deregulation underlies uncontrolled cell proliferation leading to tumour formation. Growth arrest at G0/G1 and G2/M phases or apoptosis can be initiated with DNA alterations. Many chemotherapeutic drugs are targeted at inhibiting the cell cycle in S and M phases. Several studies have reported honey treatment of cell lines leading to arrest of cells in the G0/G1 phase in bladder (T24, 253 J, RT4, and MBT-2) [32], colon (HCT-15 and HT-29) [33], and human melanoma (A375) [34] cell lines.

Multiple honey types have been tested for their in vitro effects on cell proliferation. Swellam et al. [32] examined the effect of increasing doses of pure unfractionated honey, sourced from Tokyo, Japan, in three human bladder cancer cell lines (T24, 253J, and RT4), as well as in one murine bladder cell line (MBT-2), using the 3-(4,5-dimethylthiazol-2-yl)-2,5-diphenyl tetrazolium bromide (MTT) assay. Compared to control, at 12% final concentration, honey reduced the cancer cell viability to 18% viable cells in MBT-2 and RT4 cell lines. Very few viable cells were present in T24 and 253J cells after 24 h incubation with 12% honey. A higher dose of 25% honey did not show any additional effects. The 5-bromodeoxyuridine (BrDu) labelling index also showed minimal cell viability in RT4, T24, and 253J cells in the presence of 12% honey. Flow cytometry analysis matched the BrdU labelling index and showed a significant low S phase fraction and accumulation of a large cell population behind the G1 peak in the presence of both 6% and 12% honey. Interestingly, the effect was higher in the presence of 6% honey than 12% in the 253J and RT4 cell lines [32]. Likewise the apoptotic rate in the presence of 6% honey was higher than 12%, an effect which may be related to the low cell survival rate at a higher concentration of honey.

Manuka honey (UMF 10+) was shown to inhibit cell proliferation at concentrations as low as 0.6% (*w*/*v*) in multiple cell lines (human breast cancer MCF-7, murine melanoma B16.F1, and mouse colon carcinoma CT26) in a dose and time dependent manner [35]. Using the CellTiter-Glo^®^ Luminescent cell viability assay (Promega, Madison, WI, USA), the authors found 40% inhibition after 24 h and 60% after 72 h incubation of MCF-7 cells, with 5% final concentration of honey. Similar results were shown for other cell lines, with B16.F1 viability reduced to 43% of control after 24 h and 17% after 72 h following treatment with 2.5% manuka honey. The honey treatment reduced CT26 cell viability to 30% (24 h) and 7% (72 h) to that of the control. This study also demonstrated that the antiproliferative effect of manuka honey was associated with the activation of a caspase-9-dependent apoptotic pathway [35].

Tualang honey was shown to exhibit antiproliferative effects in oral squamous and osteosarcomal cell lines [36], with complete growth inhibition at 15% final concentrations and IC50 of 4% (oral squamous cell line) and 3.5% (osteosarcomal cell line). Since honey is supersaturated with sugars, the authors also examined the effects of a mixture of glucose, fructose, and sucrose, and showed that the antiproliferative effect of honey superseded the osmolarity effects of sugars. By examining the antiproliferative activity in the presence of catalase, they also ruled out the toxic effects of hydrogen peroxide and indicated that the antiproliferative effects of honey were related to its phenolic content. Tualang honey has also been shown to be antiproliferative against several other cancer models, including human breast cancer (MCF-7 and MDA-MB-231), cervical cancer (HeLa), and leukemia (K562 and MV4-11) cell lines [37,38]. However, it did not exert cytotoxic effects on the MCF-10A, a normal breast cell line [37]. A similar selective cytotoxicity effect has been reported for gelam honey against liver cancer HepG2 cells where, compared to non-neoplastic cells [39,40], a much higher concentration of honey caused cytotoxicity of the normal cell line (25% vs. 70%).

Thyme honey has been tested for its antiproliferative effects in breast cancer (MCF-7), prostate cancer (PC3), and endometrial cancer (ishikawa) cell lines [41]. In this study, honey was shown to reduce cell viability by 10% at the highest concentration (125 ug/mL) [41]. However, the highest concentration used in this study was much lower than the concentration used by Ghasham et al. [36] and this might be the reason why the effect of honey was lower than that observed by Ghasham et al. [36]. Tsiapara et al. [41] also showed an augmentary effect of fir honey on MCF-7 cell proliferation. Although it is not understood why this honey might have exerted a stimulatory effect it may, however, be related to its nutrient (glucose, amino acids, minerals), hydrogen peroxide, and phenolic constituent content, especially quercetin, which has been reported to exert biphasic effects on cancer cells [42].

Gelam and nenas honey were tested for their antiproliferative effects using the 3-(4,5-dimethylthiazol-2-yl)-5-(3-carboxymethoxyphenyl)-2-(4-sulfophenyl)-2*H*-tetrazolium (MTS) assay in the colon cancer HT29 cell line, with an IC50 of 39 mg/mL (~3.9%) and 85 mg/mL (~8.5%), respectively reported for each honey type [43]. Their results for gelam honey were similar to the ones reported by Ghasham et al. [36]. Honey varieties differ in their phenolic content and, therefore, their antioxidant-related effects are likely to vary as well. Tualang honey has been shown to contain slightly higher phenolic and flavonoid content than gelam honey [44] and, if results are compared between Ghasham et al. [36] and Wen et al., then the slightly better IC50 for tualang honey, in an osteosarcomal cell line, compared to gelam honey, in the HT29 cell line, can be explained due its higher antioxidant content. However, the IC50 of tualang honey in an oral squamous cell line was similar to the one observed for gelam honey. For acacia honey, an IC50 of 2% (0.02 g/mL) was reported for both murine (B16-F1) and human (A375) melanoma cell lines [34]. The same honey showed an IC50 of 4.4% for 24 h and 1.9% for 48 h incubation with honey of a human prostate cancer (PC3) cell line [45]. This suggests that the effects of honey can vary between the varieties of honey, as well as the cell lines which are tested. Most studies do not provide information on the passage number of the cells tested and this might also influence the results obtained.

**Figure 2:**
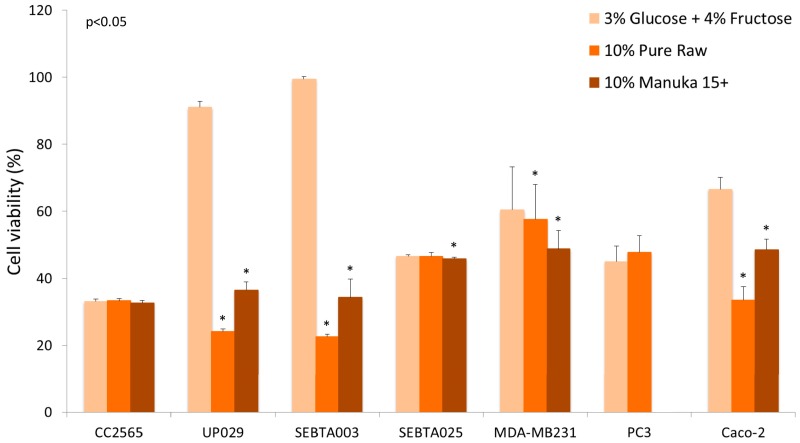
The effect of manuka 15+ honey and pure raw unprocessed honey (USA) on cell viability, using MTS assay.

Another confounding factor that might influence results when honey is tested using in vitro studies is the effect of sugars on cell proliferation. Glucose is a preferred nutrient for cancer cells and sugars present in honey have been suggested to have both mutagenic, as well as antimutagenic, effects. Wang et al. used the Ames mutagenicity assay to test the mutagenic and antimutagenic effects of seven different honeys, as well as their individual sugar components [46]. The results showed antimutagenic effects of monosaccharide sugars, as well as honeys, against food mutagen Trpp-1 (3-amino-1,4-dimethyl-5*H*-pyrido[4,3-*b*]indole), at concentrations above 100 µg/mL (0.01% *w*/*v*). Buckwheat honey was shown to be the most effective of the seven honey types which were tested [46]. While there are numerous studies which examined the antiproliferative effects of honey in cell culture, the type of honey used was often different and many studies did not compare the effect of sugars versus the effects seen for honey. In our experience, the effects of honey can vary between cell lines and also the type of honey that is used. Preliminary data showing the effects of honey on the viability of breast, prostate, colon, and several brain tumour cell lines (Figure 2) illustrates that only in some cell lines the effect of honey supersedes the effect of sugars. This disparity may be related to the phenolic content of the honey and/or the metabolism of sugars, as well as the number of glucose transporters (GLUTs) expressed by cancer cells [47].

*Legend:* Concentrations of 10% honey (manuka 15+ and pure raw unprocessed wild flower honey) were compared to a solution containing 3% glucose and 4% fructose, the equivalent amount found in honey. Four brain cell lines were studied: normal astrocyte (CC2565), grade IV glioblastoma (UP029), glioblastoma multiforme (SEBTA003, SEBTA025), as well as one breast cancer cell line (MDA-MB231), one prostate cancer cell line (PC3), and one colon cancer cell line (Caco-2). Absorbance readings were measured at 490 nm, the values being normalised to 100% of media control. The percentage of viable cells in the presence of sugars and honey were measured after 24 h incubation. A comparison of honey treatment with the sugar mixture showed a significant difference (*p* < 0.05) in some, but not all, cancer cells (Wilcoxon rank sum test with IBM SPSS Statistics for Macintosh, Version 22.0, IBM Corp. Armonk, NY, USA).

Our results are in agreement with the data obtained by Wen et al. [43] and Ghasham et al. [36], in that the effects of honey can vary between the type of honey used, as well as the cell line investigated. These results highlight that for in vitro cell culture studies, it is important to rule out the effects of sugars on cell proliferation. Secondly, if the proposed activity is suggested to be due to the antioxidant components of honey, then the antioxidant and phenolic content of honey should also be measured and correlated with the cell viability analysis. Furthermore, a higher antiproliferative effect has been shown in honeys with high phenolic content [33].

Many of the individual constituents of honey have been tested for their antiproliferative effects. Chrysin is one of the best-studied phenolics found in honey and its toxic effects have been reported against several cancer cell lines. For example, one study using chrysin at concentrations of 25 µM and 50 µM demonstrated 15% and 25% inhibition of human melanoma (A375) and 10% and 20% inhibition of murine melanoma (B16-F1) cell lines, following a 24 h treatment. The results were acquired using the MTT assay [48]. An IC50 of 50 µM was found for both human and murine melanoma cell lines after 72 h incubation. In the same study, acacia honey also caused inhibition in a time and dose dependent manner with an estimated IC50 value equal to about 0.02 g/mL, regarding both murine and human melanoma cell lines [48]. However, in the human colon cancer cell lines, maximum inhibition of HCT16 cell viability by chrysin was observed at a final concentration of 100 µM, with ~13% inhibition observed after 6 h and ~78% inhibition after 48 h of incubation [48]. Interestingly, the toxic effects were only observed in the cancer cells, while the control cell lines were not affected. In addition, cytotoxicity of chrysin has been reported against several other cancer cell lines, including breast, prostate, cervical, liver, glioblastoma, lung, liver, and pancreatic cancer [48,49,50]. Recently, chrysin’s anticancer activity has been reviewed by Kasala et al. [50], with chrysin being shown to inhibit cancer growth through modulation of phase I and phase II enzymes, induction of apoptosis, alteration of cell cycle, inhibition of angiogenesis, and invasion and metastasis. In the HCT 116 human colon cancer cell line, the luciferase assay demonstrated an increase in the activation of three transcriptional pathways (nuclear factor kappa B (NF-κB) response element, serum response element and activator protein 1 (AP-1) response element) by chrysin in a time-dependent manner [51]. This was accompanied by an increase in tumour necrosis factor-alpha (TNF-α) and tumour necrosis factor-beta (TNF-β) gene expression, and induction of cell apoptosis.

The antiproliferative effects of quercetin have been reported for HL-60 leukemia [52], MCF-7 human breast cancer [53], Caco-2 human colon adenocarcinoma [54], PC3 and DU145 prostate cancers [42,55], SCC25 oral cancer [56], ishikawa endometrial cancer [57], and SPC212 and SPC111 malignant mesothelioma cell lines [58]. In the HL-60 cell line, concentrations as low as 10 µM were found to inhibit cancer cell growth by 17% after 24 h incubation and ~53% after 96 h incubation [52]. In the case of prostate cancer cell lines, no significant effect was observed at 10 µM and 24 h incubation, however, at a higher concentration (50 µM), a significant inhibition of PC3 and DU145 cell growth was observed, but no effect was observed in LNCaP or in BG-9 normal skin fibroblast cells [55]. Some studies showed a biphasic effect of quercetin [42], a synergistic antiproliferative effect between quercetin and cisplatin also being reported [58]. Other honey constituents, i.e., caffeic acid, kaempferol, apigenin, and luteolin etc., have been tested for their anticancer properties, which has been extensively covered previously [59,60]. It is interesting to note that when tested individually, all phenolic compounds inhibit cell proliferation at concentrations which are 10- to 100-fold in excess than the amount present in honey. This suggests that either in honey, in spite of the low concentrations, synergism between these compounds leads to antiproliferative effects, or there are other cytotoxic components of honey that are yet to be studied.

### 2.2. Modulation of Growth Factor Signalling by Honey

Growth factors, through interaction with their receptors, transfer signals from outside to the inside of the cells, leading to activation of various genes. Cancer cells synthesise their own growth factors, thus evading normal growth factor responses, and become self-sufficient [61,62]. The epidermal growth factor receptor (EGFR) is a key cell surface receptor frequently up-regulated or overexpressed in cancer. Binding of ligands (epidermal growth factor (EGF), transforming growth factor-alpha (TGF-α)) to their receptors initiates several signal transduction cascades, specifically the mitogen-activated protein kinase (MAPK), protein kinase B (Akt), and c-Jun N-terminal kinase (JNK) pathways, resulting in DNA synthesis and cell proliferation [63].

Only a few studies have examined the effect of honey on growth factor signal transduction. Treatment with gelam honey (40–100 mg/mL) alone and in combination with ginger (honey 10–50 mg/mL plus ginger 3 mg/mL) led to downregulation of Kirsten rat sarcoma viral oncogene homolog (KRAS), extracellular signal-regulated kinase (ERK), and Akt genes in the colorectal cancer cell line HT29 [64]. In dermal fibroblasts, manuka honey, at a concentration of 0.1%, showed a protective effect on 2,2′-azobis (2-amidinopropane) dihydrochloride (AAPH)-induced stressed cells, by activating 5′ AMP-activated protein kinase (AMPK) phosphorylation, as well as the NrF2/ARE anti-inflammatory signalling pathway [65]. Honey treatment also upregulated the antioxidant defence and downregulated the markers of oxidative stress. The authors proposed this as a wound healing mechanism of honey [65]. Future studies should examine the effect of honey on fibroblasts in the tumour microenvironment.

While there is limited data on the effect of whole honey on growth factors and growth factor signalling, some research has shown that phenolic compounds can supress selected growth factors in vitro. Quercetin (100 µM) treatment significantly decreased EGF gene and protein expression in an endometrial cancer cell line (Ishiwaka) [57]. Treatment with 5–30 μM of caffeic acid phenethyl ester (CAPE), a compound derived from honeybee propolis, decreased the total abundance and phosphorylation of EGFR in MDA-231 breast cancer cells in a dose-dependent manner [66].

A growth inhibitory effect of ellagic acid (10 µM) has been reported for SW480 colon cancer cells through downregulation of the insulin-like growth factor 2 (IGF-II) signalling pathway [67]. Hesperetin supplementation of rats, at 20 mg/kg, has been reported to decrease 1,2-dimethylhydrazine-induced formation of angiogenic growth factors, such as vascular endothelial growth factor (VEGF), basic fibroblast growth factor (bFGF), and EGF, in experimental colon cancer [68]. Although there is little evidence on whether honey influences growth factor signalling, the above studies demonstrate the need for further research using whole honey.

### 2.3. The Apoptotic Properties of Honey

Regulation of apoptosis is critical in cancer pathogenesis, as failure to undergo apoptosis results in an uncontrolled increase in cancerous cells. Evasion of programmed cell death involves complex mechanisms with numerous molecules and processes, mainly mediated by proteolytic enzymes called caspases, which cleave specific proteins in the cytoplasm and nucleus, thus triggering cell death [69]. Mitochondria also play an important role in the regulation of certain apoptotic pathways. Many bioactive compounds, including polyphenols and vitamins found in honey, have been found to affect mitochondrial functions [70]. Targeting these processes is of great importance in order to inhibit tumour growth.

Honey has been studied in various cancer cell lines for its ability to induce apoptosis, with several mechanisms of action being suggested. The apoptotic effects of a range of Spanish honeys were observed on human peripheral blood promyelocytic leukaemia cells (HL-60) [71]. The cells were exposed for 24 and 48 h to 2.5% and 5% of three types of honey (heather, rosemary ,and polyfloral), as well as an artificial honey composed of sugars (1.8% sucrose, 7.5% maltose, 40.5% fructose, and 33.5% glucose). The apoptotic cells were identified by chromatin condensation and flow cytometry analysis, the results being compared to controls (negative control—untreated cells, and positive control of apoptosis—etoposide-treated cells). At a final concentration of 5%, all types of honey showed a significant difference in apoptotic cells when compared to the negative control, with the highest increase being achieved after 48 h of incubation with 5% heather and polyfloral honeys (about a 74% increase of apoptotic cells). The number of apoptotic cells in the honey-treated cells were also higher in comparison to the cells treated with the mixture of sugars. Furthermore, ROS production was determined using 2′,7′-dichlorodihydrofluorescein diacetate (H_2_DCFDA), the authors concluding that the investigated Spanish honeys induced apoptosis in HL-60 cells through a ROS-independent pathway [71]. An Iranian multifloral honey was found to induce apoptosis on the ACHN renal carcinoma cell lines in a time- and concentration-dependent manner, with the highest number of apoptotic cells being achieved after incubation with 20% honey for 48 h [72].

Honey has also been shown to promote apoptosis in animal models of various types of cancer. In one study [32] 100 female C3H/He mice were injected with MBT-2 mouse bladder tumour cell line cell suspension and split randomly into five groups. One group received intralesional supplementation of 0.1 mL of 12% honey for three weeks, while another group received the same treatment using 6% honey. A third group received intravenous saline instead of honey, while a fourth group received about 1.5 mL of 50% honey in drinking water. The final group was left untreated for control. The results showed a significant growth inhibitory effect of both intralesional and oral administration of honey. The authors also identified apoptotic cells in T24 human bladder cancer cells, by assessing DNA breaks during apoptosis, using the Terminal deoxynucleotidyl transferase dUTP Nick-End Labeling (TUNEL) assay [32]. The cells were treated with 6% and 12% honey for 24 h, and the results showed a higher increase in apoptotic cells when using 6% honey. However, these results were not compared with the effects of a sugar mixture or artificial honey [32].

A study investigating oral administration of honey combined with *Aloe vera* found an increase in apoptosis in Wistar rats implanted subcutaneously with a Walker 256 carcinoma cell suspension [73]. These rats received daily a 670 mL/kg dose of a solution containing honey and *Aloe vera* (500 g honey, 500 g *Aloe vera*, 30 mL ethanol), while the control group received the same amount of saline solution. The tumour cells were examined at 7, 14 and 20 days after implantation using immunohistochemistry. The researchers found that the *Aloe vera* and honey mixture increased the expression of the proapototic protein Bax, especially on day 20, while inhibiting expression of the antiapoptotic protein Bcl-2, especially in the early stages of the tumour development (day 7 and day 14). The Bax/Bcl-2 ratio in the tumours treated with the honey-*Aloe vera* combination was raised, the authors concluding that honey and *Aloe vera* can modulate tumour growth by increasing the susceptibility to apoptosis [73].

Another in vitro study on colon carcinoma cell lines (HCT-15 and HT-29) showed that a pure unfractionated Indian honey induced apoptosis through ROS production and mitochondria-dependent mechanisms, as well as via modulation of the expression of antiapoptotic and proapoptotic proteins [74]. The HCT-15 cells were treated with 3% honey for 12, 24, and 48 h, using untreated cells as a control. Using the Western blot assay, the authors analysed the expression of proteins involved in apoptosis, concluding that the protein expression was affected in a time-dependent manner with the greatest difference being observed at 48 h: with an increase in p53, caspase-3, and Bax, and a decrease in poly ADP ribose polymerase (PARP) and Bcl-2. Furthermore, the apoptotic cells were quantified using flow cytometry (YO-PRO-1 staining), the results also showing the greatest increase in apoptotic cells at 48 h: 52.60% (HCT-15) and 46.86% (HT-29). Nevertheless, the authors did not examine these effects at higher concentrations of honey and artificial honey i.e., a sugar mixture was not tested [74].

Manuka honey was shown to induce late apoptotic events in murine melanoma (B16.F1), colorectal carcinoma (CT26), and human breast cancer (MCF-7) cells in vitro, and inhibit tumour growth by increasing apoptosis in a mouse melanoma model [35]. These results were achieved by incubating for 12, 24, and 48 h with a range of manuka honey (UMF 10+) concentrations from 0.3%–5%. Paclitaxel (10 ng/mL or 50 ng/mL) was used as a positive control, while the negative control contained only media. Apoptotic cells were identified by flow cytometry, and the expression of PARP and Bcl-2 proteins were demonstrated using Western blotting. Furthermore, the effect of caspase activation, induced by manuka, on DNA fragmentation was examined by agarose gel electrophoresis. The results showed that manuka honey promotes apoptosis in a time- and dose-dependent manner through the intrinsic apoptotic pathway, which involves activation of the executioner caspase-3 by the initiator caspase-9 [35]. In addition, apoptosis was also associated with the activation of PARP, induction of DNA fragmentation, as well as decreased Bcl-2 expression [35].

Tualang honey has shown anticancer effects in breast and cervical cancer cell lines. An in vitro study on breast cancer cell lines (MDA-MB-231 and MCF-7) and HeLa cells, found that tualang honey was cytotoxic and induced cell death in a time- and dose-dependent manner [37]. The authors analysed the cancer cells by flow cytometry (fluorescence-activated cell sorting (FACS)) after incubating the cells with a IC50 dose of honey for each cell line (2.8% for MDA-MB-231, 2.4% for MCF-7, and 2.4% for HeLa cells) for 6, 24, 48 and 72 h. After staining with annexin V fluorescence antibody and propidium iodide, the cells showed maximum percentages of apoptosis after 48 h (51.2% for MDA-MB-231) and 72 h (55.6% for MCF-7 and 56.2% for HeLa cells). Furthermore, green fluorescence also indicated activation of caspase-3/7 and caspase-9 in all three types of cells after a 6 h incubation with an IC50 dose of honey, indicating apoptosis was induced by mitochondrial membrane depolarisation. When carrying out the same experiments on normal breast epithelial cell line (MCF-10A), none of these effects were observed [37].

A recent study looked at the effect of gelam honey in combination with ginger extract on colorectal cancer cell line HT29 [64]. The authors found that combining these two compounds stimulates early apoptosis by upregulating caspase-9 and IκBα genes. The mechanism of cell death was evaluated after treatment with either ginger extract, gelam honey or a combination of ginger and honey for 24 h using the Cell Death Detection ELISA PLUS 96 kit (Roche Applied Science, Mannhein, Germany). A synergistic increase in the rate of apoptosis was observed in the combined treatment group. Furthermore, RT-qPCR investigations found that the expression of the proapoptotic gene caspase-9 was upregulated in treatments with high concentrations of honey (100 mg/mL), while the combination of honey and ginger produced a similar upregulation at a much lower dose (ginger 3 mg/mL + honey 30 mg/mL). The NF-κB transcription factor inhibitor, IκBα, was highly upregulated in cancer cells treated with gelam honey (100 mg/mL), a very similar effect being observed in the cells treated with a combination of 3 mg/mL ginger and 50 mg/mL honey [64].

In addition, another study focusing on astragalus honey [75], analysed the expression of p53 and Bcl-2 genes, in a human hepatic carcinoma cell line (HepG2), human bladder carcinoma (5637), and mice fibroblast normal cell line (L929). The cells were treated with a range of honey concentrations (0.8%–6.25%), as well as a mixture of sugar (31% glucose, 39% fructose, 8% maltose, 3% sucrose) for control. Real-time PCR results showed that honey decreased the expression of Bcl-2 mRNA in HepG2 and 5637 cells, however, these results were very similar to those obtained from the cells treated with the sugar mixture. On the other hand, the expression of this gene remained unchanged in the L929 (normal) cell line. The expression of p53 gene was decreased in Hep62 cells treated with honey, while the sugar mixture caused a 2.5-fold increase when compared to the untreated control. A similar pattern was observed in the 5637 cells, honey decreasing p53 expression while the sugar control showed a two-fold increase. Interestingly, honey decreased p53 expression by half in the L929 cells, while there were no changes noticed in the cells treated with the sugar mixture.

Furthermore, the apoptotic properties of many flavonoids found in honey have been researched in various cancer cell lines. Chrysin [50], a common flavonoid of buckwheat and manuka honey, was shown to induce apoptosis in heptatocelluar (HepG2), colon (HCT116, DLD1), and rectal cancer (SW387) cell lines within the concentration range 40–100 µM [51,76,77]. In prostate (PC3), breast cancer (MDA-MB231), and lung cancer (A549) cell lines, concentrations as low as 10 µM were found to be effective [78,79,80]. In U937 leukemia cells, chrysin was shown to induce apoptosis through caspase activation and Akt inactivation [81]. Overall, the mechanisms suggested for the apoptotic activity of chrysin include (i) increasing the expression of proapoptotic proteins p53, Bax, Bad, Bak, and DR5; (ii) decreasing the levels of antiapoptotic protein (Bcl-2), regulating the p53/Bcl-2/caspase-9 signalling pathway; (iii) increasing expression of TNF-α and TNF-β, inhibiting TNF-α-mediated NF-κB activation; (iv) promoting TNF-related apoptosis-inducing ligand (TRAIL)-induced cell death, promoting AMPK activation; (v) downregulating S-phase kinase-associated protein-2 (Skp2) and low-density lipoprotein receptor-related protein 6 (LRP6) expression; and (vi) activation of caspase and inactivation of Akt [50].

Quercetin has been reported to induce apoptosis in human bladder (MB49, T24, UMUC3), cervical (CaShi, HeLa, SiHa), ovarian (OVCAR-3, TOV21G, HOSE), and breast (MCF-7) cancer cell lines [82,83,84,85]. In cervical cancer cell lines apoptosis was induced at concentrations as low as 20 µM, whereas a higher concentration range (80–100 µM) was required to significantly inhibit apoptosis in bladder, ovarian, and breast cancer cell lines. Ellagic acid is another important flavonoid found in honey and is a potent inducer of apoptosis in cancer cells [86,87,88]. This is, however, being extensively covered in another review in this issue. Kaempferol was found to induce apoptosis in both in vitro and in vivo studies in mice, with respective concentrations of 100 µM and 50 mg/kg per day for four weeks, which was shown to have a significant effect on apoptosis in bladder cancer [89,90], HT-29 colon cancer, ovarian cancer (A2780/CP70, A2780/wt, OVCAR-3), SiHa human cervical cancer, and MCF-7 breast cancer cells [91,92,93,94].

In summary, evidence to date shows a promising effect of honey and its constituents on the apoptotic pathways, especially by promoting proapoptotic protein expression and inhibiting the expression of the antiapoptotic protein Bcl-2, as well as by modulating caspase activation, p53 expression, and DNA fragmentation. Some studies found that combining honey with other natural products enhances the apoptotic effects, however, further studies investigating combinations with a wider range of antioxidant-rich products are warranted.

### 2.4. The Anti-Inflammatory and Immunomodulatory Properties of Honey

Inflammation is a biological response to injury which facilitates wound healing and plays a role in many pathological processes. Cytokines released from inflammatory cells can trigger angiogenesis or stroma proliferation, while damage caused by reactive oxygen species (ROS) to the surrounding tissues can cause tumour initiating mutations. This process is, therefore, associated with all stages of carcinogenesis, from initiation and progression, to invasion and metastasis, and has been described as the seventh hallmark of cancer [95]. The association between inflammation and cancer is further supported by epidemiological studies reporting lower incidence of colorectal, prostate, and ovarian cancer in people taking non-steroidal anti-inflammatory drugs (NSAIDs) [96,97,98]. In addition, cancer often arises near sites affected by chronic inflammatory disease, suggesting a role for anti-inflammatory treatments in cancer therapy.

The MAPK and NF-κB pathways are important components of the inflammatory response frequently dysregulated in cancers. NF-κB signalling has been identified as a major factor regulating tumour angiogenesis and invasiveness, as well as the ability of malignant cells to resist apoptosis [99]. Constitutive activation of NF-κB is shown in a wide variety of tumour types, including lung cancer, lymphoma, and breast cancer [100,101,102]. In mice, the honey constituent galangin (15 mg/kg) was shown to exert anti-inflammatory effects in asthma through inhibition of NF-κB [103]. Similarly, the flavanone chrysin, administered at 250 mg/kg bodyweight three times weekly, reduced cytosolic and mRNA levels of NF-κB and decreased the severity of diethylnitrosamine (DEN)-induced early hepatocarcinogenesis in Wistar rats [104]. CAPE treatment (50–80 μM) inhibits the activation of NF-κB in human monocytic U937 cells by preventing the translocation of the p65 unit of NF-κB and blocking the binding between NF-κB and DNA [105]. Gelam honey extract, as well as quercetin, each used at concentrations of 20, 40, 60, and 80 µg/mL for 24 h, were found to reduce the activation of both NF-κB and MAPK in a dose-dependent manner in the hamster pancreatic cell line HIT-T15 [106]. Later work from the same group found that gelam honey and quercetin (used at the same concentrations as previously) also alter JNK signalling in the same cell line, reducing the expression of the proinflammatory cytokines TNF-α, interleukin-6 (IL-6), and interleukin-1β (IL-1β) [107].

Activation of MAPK results in induction of proinflammatory cytokines including interleukin-1 (IL-1), IL-6, and TNF-α, as well as inflammatory proteins, such as C-reactive protein (CRP) and cyclooxygenase-2 (COX-2). A honey flavonoid extract (0.5 and 1 μg/mL of flavonoids) significantly inhibited the release of TNF-α and IL-1β and ROS production in lipopolysaccharide (LPS) stimulated N13 microglia [108]. Small human studies have demonstrated that natural honey treatment reduces plasma levels of key inflammatory proteins. CRP was shown to be reduced by 7% after 15 days consumption of 250 mL of water containing 75 g of natural honey [109]. A similar study [110] found that plasma prostaglandin E_2_ (PGE_2_) levels were also reduced after honey consumption. This investigation recruited 12 adult participants (nine male and three female) aged between 25 and 48 years (mean, 38 years). After a 12 h fasting period, baseline blood samples were collected before each participant consumed 250 mL of water containing 1.2 g/kg body weight of natural unprocessed honey. Blood samples were collected at 1, 2, and 3 h post-ingestion and evaluated for changes in PGE_2_. A response was seen after just 1 h with a 14% reduction in mean plasma PGE_2_ concentration. The participants were asked to continue a daily honey treatment for 15 days. On completion of the study mean plasma PGE_2_ concentration decreased by 63% [110].

Prostaglandins are produced through the action of cyclooxygenase (COX) enzymes and mediate various aspects of the inflammatory response, including fever, increased vascular permeability, and oedema [111]. An investigation using a rat paw model of inflammation (Sprague Dawley rats) found that honey treatment of 800 mg/kg led to a decrease in PGE_2_ levels, as well as a marked decrease in inflammatory activity, indicated by reduced swelling and oedema [112]. Honey methanolic and ethyl acetate extracts (180 mg/kg in 5% DMSO) produced a more profound effect than pure honey. The major phenolic compounds in both extracts were gallic acid, ellagic acid, caffeic acid, luteolin, chrysin, and quercetin, further indicating the importance of these compounds in mediating the anti-inflammatory effects of honey [112]. Induction of PGE_2_ was also suppressed by CAPE in cultured human oral epithelial cells used at 2.5 µg/mL, while higher concentrations (10–20 µg/mL) suppressed COX-2 mRNA in the same cells [113]. The authors also demonstrated that CAPE suppressed prostaglandin synthesis in a dose-dependent manner (10–100 mg/kg) and also reduced COX-2 levels in the rat carrageenan air pouch model of inflammation [113]. Expression levels of COX2 and CRP were also found to be reduced in hepatocyte-derived Chang Liver cells on co-incubation with kaempferol and a cytokine mixture in a dose-dependent manner [114]. Immunoblot analysis revealed that quercetin and kaempferol caused a dose-dependent decline in COX2 with IC50 values of 24.7 μmol/L and 17.6 μmol/L for quercetin and kaempferol, respectively. CRP protein levels were also reduced by both treatments with IC50 values of 3.7 μmol/L for quercetin and 1.8 μmol/L for kaempferol. Similar results were found using RT-PCR to evaluate mRNA expression levels, both flavonoids reduced expression of COX2 and CRP genes at all tested concentrations (5–200 μM) [114].

The pro-inflammatory cytokine interleukin-8 (IL-8) has been found to have tumorigenic and proangiogenic properties. It is a characteristic feature of many chronic inflammatory diseases and it is overexpressed in many human cancers [115]. Honey from multiple geographical sources has been shown to inhibit secretion of IL-8 in vitro, using human colon carcinoma cells (WiDr) [116]. The authors found that the phenolic content and the antioxidant activity, determined by DPPH radical scavenging assay, had no bearing on IL-8 inhibition, suggesting that other compounds must be responsible for this finding.

Other studies have demonstrated that the anti-tumour effect of honey is associated with the production of ROS following immunomodulation. In mice, bee honey has been shown to stimulate the immune system [117]. Swiss albino mice were given bee honey dissolved in distilled water at concentrations of 10, 10, or 1000 mg/100 g body weight orally (0.2 mL/mouse) every other day for four weeks. Control mice received 0.2 mL distilled water only. Several parameters were compared between these groups following inoculation of Ehrlich ascites tumour. The authors found increased macrophage phagocytic activity and T-Cell activation in the honey treated group. A human study [118] also demonstrated the immunomodulatory effect of honey consumption. Participants (seven male and three female, mean age, 31.2 years; range, 20–45 years) received a closely-controlled regular diet during a control period of two weeks, followed by a two week test period during which honey was consumed. Overnight fasting blood samples were taken at the end of the test period. The authors found that daily consumption of 1.2 g/kg body weight of honey dissolved in 250 mL of water resulted in a 50% increase in peripheral blood monocyte counts and modestly increased lymphocyte and eosinophil percentages.

Fukuda et al. [119] demonstrated that jungle honey, used at 10 mg/mL (1% *w*/*v*) with phosphate buffered saline (PBS), led to IL-1β induced activation of neutrophils, which then mediated the anti-tumour effect via release of ROS in C57BL/6 mice inoculated with Lewis Lung Carcinoma/2 (LL/2) cells. In the same manner, several types of honey (manuka, pasture, and jelly bush) were shown to induce an anti-tumour effect via immune modulation in the monocytic cell line Mono Mac6 (MM6) [120]. This group found that honey treatment caused an increased induction of IL-1β, IL-6, and TNF-α when used at 1% (*w*/*v*) compared to a no treatment control and a sugar analogue. Type II arabinogalactan proteins (APGs), compact glycosylated protein molecules, have been identified as another potential mediator of the immunomodulatory properties of New Zealand honey. AGPs purified from manuka and kanuka honey are able to stimulate the release of TNF-α from monocytic cell lines THP-1 and U937 [121].

This apparent discrepancy between the pro- and anti-inflammatory properties of honey may be due to differences in the cell lines and animal models used, as well as the honey type and composition. As outlined here, the phenolic components of honey have anti-inflammatory and antioxidant properties, which have been proposed as mechanisms for honey’s anti-tumour activity. Each variety of honey has varying amounts of these compounds altering their activity. Further studies are needed to fully elucidate the mechanism of action, particularly concerning the immunomodulatory properties of honey not associated with its antioxidant activity.

### 2.5. The Anti-Angiogenic Potential of Honey

Angiogenesis is the process of new blood vessel growth, which facilitates tissue formation by providing nutrition and oxygen to tissues. This process is important both in wound healing, as well as in the development of malignant tumours, the latter often being described as a wound that does not heal [122]. Cancer cells promote angiogenesis through generation of factors such as bFGF, TNF, and VEGF [123]. Honey is well known to promote angiogenesis in normal cells, with a varied response at different concentrations. It has been suggested that at low concentrations (0.015%–6.2%), honey has proangiogenic effects, which disappears at higher concentrations (>12.5%). Honey has been shown to decrease VEGF formation at high concentrations [124], while PGE_2_ can induce VEGF expression, leading to increase in tumour angiogenesis [125]. In the rat air pouch model of inflammation, honey has been shown to inhibit the angiogenic agents PGE_2_ and VEGF [126]. In 7,12-dimethylbenz(α)anthracene (DMBA)-induced breast cancer rats, tualang honey, at concentrations as low as 0.2 g/kg, significantly reduced the cancer growth, increased the number of apoptotic cells, and reduced VEGF levels, as well as the vasculature around the tumour [127].

Degradation of the extracellular matrix (ECM) by proteases facilitates both angiogenesis and metastasis, protease inhibitors being shown to inhibit the process of carcinogenesis [128]. Honey was found to inhibit extracellular protease and gelatinase activity in HepG2 cancer cells, however, the effects varied with the type of honey that was used [129]. In rats, feeding 300 mg/kg of propolis, a substance used by bees to fill gaps and cracks in hives, was found to inhibit bladder cancer angiogenesis in animals fed *N*-butyl-(-4-hydroxybutyl) nitrosamine [130].

Further studies using a co-culture model of endothelial and cancer cells or in vivo animal studies should identify the most effective concentrations, as well as the types of honey with high anti-angiogenic potential. Angiogenesis in wound healing and cancer have some remarkable similarities [122], the main differences being that in wound healing the process is self-limiting, while this is not the case in cancer cells. It will, therefore, be interesting to compare the effect of honey on the genes expressed in the late stages of wound repair in healthy cells with that of tumour cells in which the process continues and cells continue to grow and metastasise. Pedersen et al. [131] have identified some of these genes, which include GLI3, CRABP-II, calmodulin, galectin-7, conexxin-26, cytokeratin I, and E-cadherin.

### 2.6. The Anti-Invasive Properties of Honey

Metastasis is the most destructive feature of cancer and consists of highly complex mechanisms [132] involving a variety of molecules [133], such as matrix metalloproteinases (MMPs), integrins, cadherins, plasminogen activators, PI3Ks, Ras-like small GTPases (Rho, Rac, Cdc42), phospholipase C (PLCs), as well as focal adhesion kinases.

There is limited evidence on the effect of honey on the invasive properties of cancer cells. In an in vivo study performed using wildflower honey from Croatia before (preventative) and after (curative) tumour cell inoculation of CBA mice and Y59 rats, honey was found to have a significant anti-metastatic effect when used before tumour inoculation [134]. The mice were injected intravenously with spontaneous mammary carcinoma (MCa) cells and methylcholanthrene-induced fibrosarcoma (FS) cells, while the rats were injected with transplantable anaplastic colon adenocarcinoma (ACa) cells. The mice received an oral dose of 2 g/kg, while the dose for the rats was 1 g/kg of wildflower honey from Croatia, for 10 days before and after treatment. Interestingly, when honey was administered two days after tumour cell inoculation, there was no effect on the formation of tumour nodules in mice, while in rats an even more enhanced tumour growth was observed. The authors associated this effect with activation of the immune system (specifically macrophages) by honey when used preventatively.

Matrix metalloproteinases are important contributors to the invasive and metastatic properties of cancer cells, their expression (especially MMP-2 and MMP-9) being elevated in a range of carcinomas [135,136,137]. A limited number of studies have examined the effects of honey on MMP expression or activity in cancer. In one study, Polish honeys were shown to decrease MMP-2 and MMP-9 activity in the glioblastoma cell line U87MG [138]. The cells were treated for 24 h with a concentration of 5% of four types of honey: buckwheat, multifloral light, willow (*Salix spp.*), and multifloral dark. The MMP-2 and MMP-9 activity was assessed using gelatin zymography, the gels being stained with Coomassie brilliant blue. Although the results show significant differences between the honey-treated cells and the control, it is not clear what the researchers used as a control. Thus, for the future it would be interesting to see whether these results are consistent with results obtained from cells treated with a sugar mixture containing the equivalent of sugar found in these types of honey. The authors also observed that the strongest inhibition of MMP-2 and MMP-9 expression occurred in the cells treated with the honey with the highest phenolic content. Furthermore, the amount of lead and cadmium was also analysed, the authors suggesting a correlation between these and the anti-metastatic activity of honey, as the least inhibition of MMP-2 and MMP-9 expression was noticed in the cells treated with the honey containing the highest amount of cadmium. In HT29 colon cancer cells, combined treatment with gelam honey and ginger reduced the expression of various genes involved in the Ras/ERK and PI3K/AKT pathways, which could have a downstream effect on the expression of certain MMPs involved in tumour invasion, such as MMP-2, MMP-9, or MMP-10 [64]. The results showed that the most effective dose in the downregulation of ERK was a combination of 3 mg/mL ginger and 50 mg/mL gelam honey, while for inhibiting AKT expression a combination of 3 mg/mL ginger and 30 mg/mL honey showed the best results.

There is an increasing amount of evidence in the literature on the anti-metastatic properties of various flavonoids and phenolic compounds found in honey, inhibiting both invasion and migration. Chrysin at a concentration range of 5–50 µM has been shown to downregulate the expression of MMP-2 in glioblastoma (50 µM) [139], MMP-9 in gastric cancer (>20 µM) [140] and MMP-10 in MCF-7 breast cancer cell line (>5 µM) [141], thus inhibiting the degradation of ECM and the initiation of the epithelial-mesenchymal transition. On the other hand, Spoerlein et al. found that chrysin, and a chrysin-Cu(II) complex, only slightly decreased MMP-2 expression, while having no significant effect on MMP-9 expression in melanoma cells [142].

CAPE (5–40 µM) was shown to decrease the enzymatic activity of MMP-2 in a dose-dependent manner in the oral cancer cell line SCC-9. Used at 40 µM, CAPE decreased MMP-2 enzymatic activity by 57% following 24 h treatment [143]. Other studies demonstrated that caffeic acid (50 μmol/L) caused inhibition of migration by suppressing the motility of oral carcinoma (SCC-9) and colon adenocarcinoma (A549) cells [144,145] or by suppressing the activity of voltage-gated sodium channels in breast cancer [146]. A review by Kuo et al. [147] describes various mechanisms of CAPE to induce cell cycle arrest and apoptosis in oral cancer, including inhibiting Akt signalling and regulating MMP activity, while another review by Lin et al. [148] describes the anticancer effects of CAPE, especially the inhibition of Akt signalling, in prostate cancer.

Moreover, gallic acid has also been shown to downregulate the AKT/small GTPase signalling pathway [149,150,151,152,153] and MMP (MMP-2, MMP-9) expression [151,154] in gastric carcinoma, osteosarcoma, glioma, cervical and breast cancer. In the human breast cancer cell line MCF-7, concentrations of gallic acid as low as 1 µM inhibited MMP-2 expression [154], however, higher concentrations of 2 µM have been reported for gastric adenocarcinoma cells [149] and 20 µM for osteocarcinoma cells [151]. In the latter cell lines, inhibition of MMP-2, MMP-7, and MMP-9 was reported. Chlorogenic acid, a phenolic compound often found in Malaysian honey [12], was shown to have a strong inhibitory effect at concentrations above 30 µg/mL (~84 µM) on the expression of MMP-9 in a human hepatocellular carcinoma cell line (Hep3B) [155]. Furthermore, a recent in silico study, using Discovery Studio^®^ Version 3.1 (Accelrys, San Diego, CA, USA) simulation software, found various honey constituents to be potential MMP-2 and MMP-9 inhibitors [156]. It is also worth noting that other apitherapeutic products, such as bee venom, also have anti-metastatic properties by downregulating MMP expression through inhibition of the PI3K/Akt/mTOR pathway [157].

Overall, honey constituents are shown to lower the expression of MMP-2 and MMP-9 in a range of cancer cell lines, however, currently there is limited evidence on the effect of honey on the metastatic properties of cancer. The suitable concentration range, as well as the type of honey which is likely to have high inhibitory effects, need to be identified.

A summary of the effects of honey demonstrated using in vitro, in vivo and human studies are shown in Table 1 and Table 2.

## 3. Honey for Chemoprevention and as an Adjunct to Anticancer Drugs

The negative side effects of chemotherapeutic treatments can severely impact the quality of life for patients. Therefore, therapies which can prevent progression to malignancy, reduce the required dosage of conventional drugs, or lessen the severity of adverse effects are of considerable benefit. In an experimental model of carcinogenesis, tualang and manuka honey administered to rats at a dose of 1 g/kg body weight, a week before the induction of breast carcinogenesis with *N*-methyl-*N*-nitrosourea, significantly inhibited tumour development, with manuka honey being more effective than tualang honey [158]. Another study also reported a reduction in 7,12-dimethylbenz(*α*)anthracene (DMBA)-induced breast cancer tumour size, when tualang honey was supplemented at a dose of 0.2–2 g/kg body weight for 150 days [127]. Although the mean apoptotic index in this study was not found to be significantly different between the control and honey-supplemented rats, several other cancer-related factors, including tumour size, grade, angiogenesis and VEGF levels, were reduced. Propolis, caffeic acid, wild flower honey, royal jelly, and bee venom were examined for their effects in a transplantable murine tumour model [159]. All interventions were tested for their effectiveness when given before or after the tumour mammary cell inoculations. A significant antimetastatic effect of honey was observed when 2 g/kg honey was given every day for 10 days prior to tumour cell inoculation. Treatment with honey after the tumour cell inoculation, led to an increase in the number of tumour nodules in the lungs, but it was not statistically significant. Both propolis (150 mg/kg) and caffeic acid (150 mg/kg) showed a similar significant protective effect when given before or after the tumour cell inoculation. Royal jelly failed to show a protective effect when given before or after tumour cell inoculation, but it showed a significant inhibition when injected synchronously with the tumour cells.

In mice, both oral and intralesional injection of honey reduced the bladder tumour volume size in MBT-2 tumour cell suspension-induced cancer. For oral administration, 50% honey solution was used and for intralesional injection, 12% and 6% solutions were used. What is interesting about this study is that the mice were allowed to develop a tumour first and honey intervention was only introduced after the tumour volume reached 100–150 mm^3^ [32]. Further studies like this should be undertaken to confirm whether honey can be used to reduce, or even treat, pre-existing tumours. Different types of tumours, varieties of honey, and their therapeutic concentration need to be tested. If findings similar to the ones reported by Swellam et al. [32] can be shown for other cancer types, honey will have tremendous clinical implications for the treatment of cancer.

Chemotherapeutic drugs indiscriminately target both healthy and cancer cells and are, therefore, toxic to the biological system. In cell culture studies, the cytotoxic effect of the anticancer drug 4-hydroxytamoxifen at a final concentration of 10 µM was reduced by 1% tualang honey in a non-tumour MCF-10A breast cell line. Tualang honey enhanced the Tamoxifen-induced anticancer activity in both the oestrogen receptor-(ER-)responsive (MCF-7) and ER-nonresponsive (MDA-MB-231) human breast cancer cell lines, but dampened the toxic effect of the drug in the MCF-10A cells, suggesting that tualang honey increases the effectiveness of Tamoxifen against the cancer cells, as well as protects healthy cells from the drug’s toxic effects [160,161]. Honey was also found to increase the expression of DNA repair proteins Ku70 and Ku80 in MCF-10A cells [160].

Furthermore, manuka honey has been shown to reduce the toxicity of the anticancer drug Paclitaxel in mice [35]. In this study C57BL/6 mice were administered biweekly intravenous injections of either 50% manuka suspension, 10 mg/kg paclitaxel, or a combination of both treatments. Saline (100 mL) was used as a control. At days 20 and 24 after treatment, the apoptotic cells present in the tumours were identified using caspase-3 immunohistochemical assay. The number of caspase-3-positive cells was the highest in the tumours from the mice treated with the combination of manuka with paclitaxel, suggesting that administration of manuka honey together with the standard chemotherapeutic drug may decrease its cytotoxic side effects.

In another study, a mixture of honey bee products (honey, royal jelly, pollen grains) at concentrations equivalent to the recommended daily intake in humans, ameliorated the genotoxic effects of 20 mg/kg body weight of the anticancer drug cyclophosphamide in mice [162]. One tablespoon of sunflower honey taken for 14 days reduced menopausal complaints in breast cancer patients receiving Tamoxifen and aromatase inhibitors [163]. However, the group receiving aromatase inhibitors also showed an increase in oestrogen levels, raising some concerns on the use of honey for patients receiving this treatment.

Cisplatin, another common chemotherapeutic drug, is known for its nephrotoxic side effects. In rats, oral administration of crude honey at 500 mg/kg per day for one week prior to and three days after the administration of cisplatin, reduced the nephrotoxicity of the drug and the mechanism was described through suppression of NF-kB activation by honey [164].

Honey has been suggested to have potential benefits in radiation-induced mucositis and radiotherapy-induced skin reactions in chemotherapy patients [165]. Febrile neutropenia, a side effect of chemotherapy, was shown to be reduced in leukaemia patients by Life-Mel honey (Express Honey, Tzuf Globus, Israel) (5 g/day for five days) [166] and unprocessed raw honey (2.5 g/kg, twice a week for 12 weeks) [167]. Oral mucositis, a side effect of radiotherapy and chemotherapy, remains a challenge to manage. A systematic review by Song et al. [168] and its further evaluation by Van Den Wyngaer [169], suggested a promising effect of honey on mucositis, but warranted the need for further clinical trials to confirm its benefits.

## 4. Safety

Honey has been used for centuries as a source of nutrition and topical wound treatment with no reports of adverse effects from the majority of varieties. Gastrointestinal upset and a case of hepatitis [170] has been reported following consumption of honey derived from Rhododendron flowers, colloquially known as “mad honey”, however, such incidences are rare and restricted to specific sources. Recently, the safety of honey as a medicinal agent has been demonstrated in a study conducted by Fernandez-Cabezudo et al. [35]. The authors treated tumour-bearing mice with an intravenous injection of manuka honey for three weeks with no indication of any side effects when haematological and clinical chemistry parameters were evaluated.

## 5. Conclusions

Overall, evidence from cell culture and animal studies look promising for honey in terms of chemo prevention, as well as an adjunct therapy to cancer drugs. Honey is readily available, inexpensive, easy to administer, and poses minimal risk of adverse side effects. However, the specific composition and properties of honey and its anticancer effects vary with the nectar source, honey bee species, climate, geographical region, extent of processing, as well as packaging and storage [6]. Efficacy after in vivo supplementation will depend on the extent to which phenolic levels are raised in the blood, and also on the active constituents reaching the tumour site at sufficient concentrations. Studies have demonstrated that phenolics sourced from honey are bioavailable in humans [26], however, it remains to be seen whether the concentration achieved in blood is sufficient to intercept the cancer processes in vivo. Findings from the in vitro and in vivo animal studies mentioned in this review suggest a positive impact of honey on several aspects of cancer. There are, however, several modifiers of metastasis which are yet to be studied for the effects of honey. For example, cancer-associated fibroblasts play an important role in tumour progression; honey has been shown to increase their proliferation during wound healing and was also reported to provide protection from radiation-induced damage to human diploid fibroblasts [171]. Therefore, further studies should examine the effects of honey on cancer-associated fibroblasts. The effect of honey on extracellular-degrading proteases i.e., matrix metalloproteinases, of which fibroblasts are an important source, has not been studied extensively to date. Honey’s effects on the growth signalling pathways and on the invasive properties of cancer cells are also under-researched areas. Direct injection of honey into a tumour appears to be an attractive option, however, further research is needed to evaluate the overall effectiveness of this approach. Although cancer cells feed on sugars, the high sugar content of honey, if injected directly into the tumour site, may influence the tumour stroma through the osmolarity effect. It would also be interesting to examine if such an approach could prevent neoplastic lesions from becoming invasive, as well as to measure the length of time during which these effects are sustained after treatment. Therefore, further mechanistic studies are needed to verify the anticancer potential of honey before recommendations for its use in clinical trials can be made.

## Figures and Tables

**Table 1:** The in vitro effects of a variety of honey types on a range of cancer cell lines.

In Vitro Effects of Honey	Cell Line	Honey Type	Reference
**Antiproliferative effects of honey**			
Decreases cell viability	T24, 253 J, RT4, MBT-2	Pure unfractionated (Tokyo)	[32]
	MCF-7, PC3, ishikawa	Thyme (Greece)	[41]
	MDA-MB-231, MCF-7, HeLa	Tualang (Malaysia)	[37]
Inhibits cell proliferation	MCF-7, B16.F1, CT26	Manuka UMF 10+ (New Zealand)	[35]
	HOS (CRL-1543)	Tualang (Malaysia)	[36]
	OSCC (CRL-1623)	Tualang (Malaysia)	[36]
	K562, MV4-11	Tualang (Malaysia)	[38]
	HepG2	Gelam (Malaysia)	[40]
	HT29	Gelam and Nenas (Malaysia)	[43]
	B16-F1, A375	Acacia (Unspecified)	[34]
	PC3	Acacia (Pakistan)	[45]
**Apoptotic properties of honey**			
Induces apoptosis	ACHN	Multifloral (Iran)	[72]
	T24	Pure unfractionated (Tokyo)	[32]
	HCT-15, HT-29	Pure unfractionated (India)	[74]
Induces apoptosis via ROS-independent pathway	HL-60	Heather, rosemary and polyfloral (Spain)	[71]
Induces apoptosis via mitochondrial membrane depolarisation	MDA-MB-231, MCF-7, HeLa	Tualang (Malaysia)	[37]
Increases caspase expression or activation	B16.F1, MCF-7, CT26	Manuka UMF 10+ (New Zealand)	[35]
	MDA-MB-231, MCF-7, HeLa	Tualang (Malaysia)	[37]
	HCT-15, HT-29	Pure unfractionated (India)	[74]
	HT29	Gelam (Malaysia)	[64]
Increases expression of pro apoptotic proteins	HCT-15, HT-29	Pure unfractionated (India)	[74]
Decreases expression of anti-apoptotic proteins	B16.F1, MCF-7, CT26	Manuka UMF 10+ (New Zealand)	[35]
	HCT-15, HT-29	Pure unfractionated (India)	[74]
	HepG2, 5637	Astragalus (Iran)	[75]
**Growth factor modulation by honey**			
Downregulation of RAS/ERK and PI3K/Akt signalling	HT29	Gelam (Malaysia)	[64]
**Anti-inflammatory and immune-modulatory effects of honey**			
Reduce activation of NF-κB and MAPK	HIT-T15	Gelam (Malaysia)	[106]
Reduces expression of pro-inflammatory cytokines	HIT-T15	Gelam (Malaysia)	[107]
Inhibits expression of IL-8	WiDr	Monofloral (D. longan, L. chinensis, C. maxima and A. formosana) and one multifloral honey (Taiwan)	[116]
Increases expression of proinflamatory cytokines IL-1β, Il-6, TNF-α	MM6	Manuka, Pasture (New Zealand) and Jelly bush (Australia)	[120]
**Anti-angiogenic effects of honey**			
Inhibits extracellular protease and gelatinase activity	HepG2	Unspecified (Saudi Arabia and Egypt)	[129]
**Anti-invasive effects of honey**			
Decreases MMP-2 and MMP-9 activity	U87MG	Buckwheat, Multifloral light, Willow and Multifloral dark (Poland)	[138]
	HT29	Gelam (Malaysia)	[64]

**Table 2:** The in vivo effects of a variety of hone types on a range of cancer models, including human studies investigating the effects of honey on biomarkers of inflammation.

In Vivo Effects of Honey	Animal Model	Honey Type	Reference
**Apoptotic effects of honey**			
Tumour growth inhibition	MBT-2 Mouse bladder tumour (C3H/He mice)	Pure unfractionated (Tokyo) (IL, Oral)	[32]
	Syngeneic mouse melanoma model (C57BL/6 mice)	Manuka UMF 10+ (IV)	[35]
	Lewis Lung Carcinoma/2 (C57BL/6 mice)	Jungle (Nigeria) (IP)	[119]
Increases expression of Bax, inhibits expression of Bcl-2, Increases Bax/Bcl-2 ratio	Walker 256 carcinoma (Wistar rats)	Unspecified (Oral)	[73]
**Anti-inflammatory effects of honey**			
Reduces swelling and oedema with decreased PGE_2_ levels	Rat paw oedema model (Sprague Dawley rats)	Gelam (Malaysia) (IP)	[112]
Increases macrophage phagocytic activity, activates T-cells	Ehrlich ascites tumour (Swiss albino mince)	Bee honey (Oral)	[117]
IL-1β induced neutrophil activation. Increased ROS	Lewis Lung Carcinoma/2 (C57BL/6 mice)	Jungle (Nigeria) (IP)	[119]
**Anti-angiogenic effects of Honey**			
Reduces tumour growth, increases the number of apoptotic cells. Reduces VEGF, decreases vasculature around the tumour	DMBA-induced breast cancer (Sprague-Dawley rats)	Tualang (Malaysis)	[127]
**Anti-invasive effects of Honey**			
Antimetastatic effects when used preventatively	Mammary carcinoma (CBA mice) Methylcholanthrene-induced fibrosarcoma (CBA mice) Anaplastic colon adenocarcinoma (Y59 rats)	Wildflower (Croatia)	[134]
**Human Studies**			
**Inflammation**			
Reduces CRP	Eight subjects	Natural honey	[109]
Reduces PGE_2_	Twelve subjects	Natural unprocessed honey	[110]
Increases peripheral blood monocyte, lymphocyte and eosinophil count	Ten subjects	Natural honey	[118]

IV, intravenous injection; IP, intraperitoneal injection; IL, intralesional injection.

## References

[1] Siegel R.L., Miller K.D., Jemal A. (2016). Cancer Statistics, 2016. CA Cancer J. Clin..

[2] Anand P., Kunnumakkara A.B., Sundaram C., Harikumar K.B., Tharakan S.T., Lai O.S., Sung B., Aggarwal B.B. (2008). Cancer is a preventable disease that requires major lifestyle changes. Pharm. Res..

[3] Irigaray P., Newby J.A., Clapp R., Hardell L., Howard V., Montagnier L., Epstein S., Belpomme D. (2007). Lifestyle-related factors and environmental agents causing cancer: An overview. Biomed. Pharmacother..

[4] Alvarez-Suarez J.M., Tulipani S., Díaz D., Estevez Y., Romandini S., Giampieri F., Damiani E., Astolfi P., Bompadre S., Battino M. (2010). Antioxidant and antimicrobial capacity of several monofloral Cuban honeys and their correlation with color, polyphenol content and other chemical compounds. Food Chem. Toxicol..

[5] Alvarez-Suarez J.M., González-Paramás A.M., Santos-Buelga C., Battino M. (2010). Antioxidant characterization of native monofloral Cuban honeys. J. Agric. Food Chem..

[6] Da Silva P.M., Gauche C., Gonzaga L.V., Costa A.C.O., Fett R. (2016). Honey: Chemical composition, stability and authenticity. Food Chem..

[7] Pasini F., Gardini S., Marcazzan G.L., Caboni M.F. (2013). Buckwheat honeys: Screening of composition and properties. Food Chem..

[8] Liou G.-Y., Storz P. (2010). Reactive oxygen species in cancer. Free Radic. Res..

[9] Coussens L.M., Werb Z. (2002). Inflammation and cancer. Nature.

[10] Gorjanović S.Ž., Alvarez-Suarez J.M., Novaković M.M., Pastor F.T., Pezo L., Battino M., Sužnjević D.Ž. (2013). Comparative analysis of antioxidant activity of honey of different floral sources using recently developed polarographic and various spectrophotometric assays. J. Food Compos. Anal..

[11] Alvarez-Suarez J.M., Giampieri F., González-Paramás A.M., Damiani E., Astolfi P., Martinez-Sanchez G., Bompadre S., Quiles J.L., Santos-Buelga C., Battino M. (2012). Phenolics from monofloral honeys protect human erythrocyte membranes against oxidative damage. Food Chem. Toxicol..

[12] Kassim M., Achoui M., Mustafa M.R., Mohd M.A., Yusoff K.M. (2010). Ellagic acid, phenolic acids, and flavonoids in Malaysian honey extracts demonstrate in vitro anti-inflammatory activity. Nutr. Res..

[13] Bertoncelj J., Dobersek U., Jamnik M., Golob T. (2007). Evaluation of the phenolic content, antioxidant activity and colour of Slovenian honey. Food Chem..

[14] Vallianou N.G., Gounari P., Skourtis A., Panagos J., Kazazis C. (2014). Honey and its anti-inflammatory, anti-bacterial and anti-oxidant properties. Gen. Med..

[15] Van den Berg A.J.J., van den Worm E., van Ufford H.C.Q., Halkes S.B.A., Hoekstra M.J., Beukelman C.J. (2008). An in vitro examination of the antioxidant and anti-inflammatory properties of buckwheat honey. J. Wound Care.

[16] Ahmed S., Othman N. (2013). Review of the medicinal effects of tualang honey and a comparison with manuka honey. Malays. J. Med. Sci..

[17] Sergiel I., Pohl P., Biesaga M. (2014). Characterisation of honeys according to their content of phenolic compounds using high performance liquid chromatography/tandem mass spectrometry. Food Chem..

[18] Chan C.W., Deadman B.J., Manley-Harris M., Wilkins A.L., Alber D.G., Harry E. (2013). Analysis of the flavonoid component of bioactive New Zealand mānuka (*Leptospermum scoparium*) honey and the isolation, characterisation and synthesis of an unusual pyrrole. Food Chem..

[19] Moniruzzaman M., Sulaiman S.A., Gan S.H. (2016). Phenolic acid and flavonoid composition of malaysian honeys. J. Food Biochem..

[20] Khalil M.I., Alam N., Moniruzzaman M., Sulaiman S.A., Gan S.H. (2011). Phenolic acid composition and antioxidant properties of Malaysian honeys. J. Food Sci..

[21] Wieczorek J., Pietrzak M., Pomianowski J., Wieczorec Z. (2014). Honey as a source of bioactive compounds. Pol. J. Nat. Sci..

[22] Yao L., Datta N., Tomás-Barberán F.A., Ferreres F., Martos I., Singanusong R. (2003). Flavonoids, phenolic acids and abscisic acid in Australian and New Zealand Leptospermum honeys. Food Chem..

[23] Ismail N.I., Kadir M.R.A., Zulkifli R.M. (2015). Isolation and identification of potential antineoplastic bioactive phenolic compounds in malaysian honeys. J. Appl. Pharm. Sci..

[24] Moniruzzaman M., An C.Y., Rao P.V., Hawlader M.N.I., Azlan S.A.B.M., Sulaiman S.A., Gan S.H. (2014). Identification of phenolic acids and flavonoids in monofloral honey from bangladesh by high performance liquid chromatography: Determination of antioxidant capacity. Biomed. Res. Int..

[25] Lachman J., Hejtmánková A., Sýkora J., Karban J., Orsák M., Rygerová B. (2010). Contents of major phenolic and flavonoid antioxidants in selected Czech honey. Czech J. Food Sci..

[26] Schramm D., Karim M., Schrader H., Holt R., Cardetti M., Keen C. (2003). Honey with high levels of antioxidants can provide protection in healthy human subjects. J. Agric. Food Chem..

[27] Jaganathan S.K., Balaji A., Vellayappan M.V., Asokan M.K., Subramanian A.P., John A.A., Supriyanto E., Razak S.I., Marvibaigi M. (2015). A review on antiproliferative and apoptotic activities of natural honey. Anticancer Agents Med. Chem..

[28] Subramanian A.P., John A.A., Vellayappan M.V., Balaji A., Jaganathan S.K., Mandal M., Supriyanto E. (2016). Honey and its phytochemicals: Plausible agents in combating colon cancer through its diversified actions. J. Food Biochem..

[29] Erejuwa O., Sulaiman S., Wahab M. (2014). Effects of honey and its mechanisms of action on the development and progression of cancer. Molecules.

[30] Allan J.M., Travis L.B. (2005). Mechanisms of therapy-related carcinogenesis. Nat. Rev. Cancer.

[31] Hanahan D., Weinberg R.A. (2011). Hallmarks of cancer: The next generation. Cell.

[32] Swellam T., Miyanaga N., Onozawa M., Hattori K., Kawai K., Shimazui T., Akaza H. (2003). Antineoplastic activity of honey in an experimental bladder cancer implantation model: In vivo and in vitro studies. Int. J. Urol..

[33] Jaganathan S.K., Mandal M. (2009). Honey Constituents and their apoptotic effect in colon cancer cells. J. ApiProd. ApiMed. Sci..

[34] Pichichero E., Cicconi R., Mattei M., Muzi M.G., Canini A. (2010). Acacia honey and chrysin reduce proliferation of melanoma cells through alterations in cell cycle progression. Int. J. Oncol..

[35] Fernandez-Cabezudo M.J., El-Kharrag R., Torab F., Bashir G., George J.A., El-Taji H., Al-Ramadi B.K. (2013). Intravenous administration of manuka honey inhibits tumor growth and improves host survival when used in combination with chemotherapy in a melanoma mouse model. PLoS ONE.

[36] Ghashm A.A., Othman N.H., Khattak M.N., Ismail N.M., Saini R. (2010). Antiproliferative effect of Tualang honey on oral squamous cell carcinoma and osteosarcoma cell lines. BMC Complement. Altern. Med..

[37] Fauzi A.N., Norazmi M.N., Yaacob N.S. (2011). Tualang honey induces apoptosis and disrupts the mitochondrial membrane potential of human breast and cervical cancer cell lines. Food Chem. Toxicol..

[38] Nik Man N.M.K., Hassan R., Ang C.Y., Abdullah A.D., Mohd Radzi M.A.R., Sulaiman S.A. (2015). Antileukemic effect of tualang honey on acute and chronic leukemia cell lines. Biomed. Res. Int..

[39] Jubri Z., Narayanan N., Karim N., Ngah W. (2012). Antiproliferative activity and apoptosis induction by gelam honey on liver cancer cell line. Int. J. Appl. Sci. Technol..

[40] Abu N.M., Salleh M.A.M., Radzman N.H.M., Ismail W.I.W., Yusof R.M., Hassan H.F. (2013). Insulin Sensitivity Enhancement of the Mixture of Tinospora Crispa and Gelam (Melaleuca Cajuputi) Honey and Its Antiproliferative Activity on Hepatocellular Carcinoma, HepG2: A Preliminary Study. J. Med. Res. Dev..

[41] Tsiapara A.V., Jaakkola M., Chinou I., Graikou K., Tolonen T., Virtanen V. (2009). Bioactivity of Greek honey extracts on breast cancer (MCF-7), prostate cancer (PC-3) and endometrial cancer (Ishikawa) cells: Profile analysis of extracts. Food Chem..

[42] Van der Woude H., Gliszczyńska-Swigło A., Struijs K., Smeets A., Alink G.M., Rietjens I.M.C.M. (2003). Biphasic modulation of cell proliferation by quercetin at concentrations physiologically relevant in humans. Cancer Lett..

[43] Wen C.T.P., Hussein S.Z., Abdullah S., Karim N.A., Makpol S., Yusof Y.A.M. (2012). Gelam and Nenas Honeys Inhibit Proliferation of HT 29 Colon Cancer Cells by Inducing DNA Damage and Apoptosis while Suppressing Inflammation. Asian Pac. J Cancer Prev..

[44] Kishore R.K., Halim A.S., Syazana M.S.N., Sirajudeen K.N.S. (2011). Tualang honey has higher phenolic content and greater radical scavenging activity compared with other honey sources. Nutr. Res..

[45] Aliyou M., Odunola O., Farooq A., Mesaik A., Choudhary M., Fatima B., Qureshi T.A., Erukainure O.L. (2012). Acacia honey modulates cell cycle progression, pro-inflammatory cytokines and calcium Ions secretion in PC-3 cell line. J. Cancer Sci. Ther..

[46] Wang X.-H., Andrae L., Engeseth N.J. (2002). Antimutagenic effect of various honeys and sugars against Trp-p-1. J. Agric. Food Chem..

[47] Medina R.A., Owen G.I. (2002). Glucose transporters: Expression, regulation and cancer. Biol. Res..

[48] Khoo B.Y., Chua S.L., Balaram P. (2010). Apoptotic effects of chrysin in human cancer cell lines. Int. J. Mol. Sci..

[49] Xuan H., Zhang J., Wang Y., FU C., Zhang W. (2016). Anti-tumor activity evaluation of novel chrysin-organotin compound in MCF-7 cells. Bioorg. Med. Chem. Lett..

[50] Kasala E.R., Bodduluru L.N., Madana R.M., Athira K.V., Gogoi R., Barua C.C. (2015). Chemopreventive and therapeutic potential of chrysin in cancer: Mechanistic perspectives. Toxicol. Lett..

[51] Ronnekleiv-Kelly S., Nukaya M., Diaz-Diaz C., Megna B., Carney P., Geiger P., Kennedy G.D. (2016). Aryl hydrocarbon receptor-dependent apoptotic cell death induced by the flavonoid chrysin in human colorectal cancer cells. Cancer Lett..

[52] Kang T.B., Liang N.C. (1997). Studies on the inhibitory effects of quercetin on the growth of HL-60 leukemia cells. Biochem. Pharmacol..

[53] Choi J.A., Kim J.Y., Lee J.Y., Kang C.M., Kwon H.J., Yoo Y.D., Kim T.W., Lee Y.S., Lee S.J. (2001). Induction of cell cycle arrest and apoptosis in human breast cancer cells by quercetin. Int. J. Oncol..

[54] Salucci M., Stivala L.A., Maiani G., Bugianesi R., Vannini V. (2002). Flavonoids uptake and their effect on cell cycle of human colon adenocarcinoma cells (CaCO_2_). Br. J. Cancer.

[55] Nair H.K., Rao K.V.K., Aalinkeel R., Mahajan S., Chawda R., Schwartz S.A. (2004). Inhibition of prostate cancer cell colony formation by the flavonoid quercetin correlates with modulation of specific regulatory genes. Clin. Diagn. Lab. Immunol..

[56] Elattar T.M., Virji A.S. (2000). The inhibitory effect of curcumin, genistein, quercetin and cisplatin on the growth of oral cancer cells in vitro. Anticancer Res..

[57] Kaneuchi M., Sasaki M., Tanaka Y., Sakuragi N., Fujimoto S., Dahiya R. (2003). Quercetin regulates growth of Ishikawa cells through the suppression of EGF and cyclin D1. Int. J. Oncol..

[58] Demiroglu-Zergeroglu A., Basara-Cigerim B., Kililc E., Yanikkaya G. (2010). The investigation of effects of quercetin and Its combination with cisplatin on malignant mesothelioma cells In vitro. J. Biomed. Biotechnol..

[59] Jaganathan S.K., Mandal M. (2009). Antiproliferative effects of honey and of its polyphenols: A review. J. Biomed. Biotechnol..

[60] Lin Y., Shi R., Wang X., Shen H.-M. (2008). Luteolin, a flavonoid with potential for cancer prevention and therapy. Curr. Cancer Drug Targets..

[61] Aaronson S.A. (1991). Growth factors and cancer. Science.

[62] Witsch E., Sela M., Yarden Y. (2010). Roles for growth factors in cancer progression. Physiology.

[63] Wagner E.F., Nebreda Á.R. (2009). Signal integration by JNK and p38 MAPK pathways in cancer development. Nat. Rev. Cancer.

[64] Tahir A.A., Sani N.F.A., Murad N.A., Makpol S., Ngah W.Z.W., Yusof Y.A.M. (2015). Combined ginger extract & Gelam honey modulate Ras/ERK and PI3K/AKT pathway genes in colon cancer HT29 cells. Nutr. J..

[65] Alvarez-Suarez J.M., Giampieri F., Cordero M., Gasparrini M., Forbes-Hernández T.Y., Mazzoni L., Afrin S., Beltrán-Ayala P., González-Paramás A.M., Santos-Buelga C. (2016). Activation of AMPK/Nrf2 signalling by Manuka honey protects human dermal fibroblasts against oxidative damage by improving antioxidant response and mitochondrial function promoting wound healing. J. Funct. Foods.

[66] Wu J., Omene C., Karkoszka J., Bosland M., Eckard J., Klein C.B., Frenkel K. (2011). Caffeic acid phenethyl ester (CAPE), derived from a honeybee product propolis, exhibits a diversity of anti-tumor effects in pre-clinical models of human breast cancer. Cancer Lett..

[67] Narayanan B.A., Re G.G. (2001). IGF-II down regulation associated cell cycle arrest in colon cancer cells exposed to phenolic antioxidant ellagic acid. Anticancer Res..

[68] Nalini N., Aranganathan S., Kabalimurthy J. (2012). Chemopreventive efficacy of hesperetin (citrus flavonone) against 1,2-dimethylhydrazine-induced rat colon carcinogenesis. Toxicol. Mech. Methods.

[69] Elmore S. (2007). Apoptosis: A review of programmed cell death. Toxicol. Pathol..

[70] Forbes-Hernández T.Y., Giampieri F., Gasparrini M., Mazzoni L., Quiles J.L., Alvarez-Suarez J.M., Battino M. (2014). The effects of bioactive compounds from plant foods on mitochondrial function: A focus on apoptotic mechanisms. Food Chem. Toxicol..

[71] Morales P., Haza A.I. (2013). Antiproliferative and apoptotic effects of spanish honeys. Pharmacogn. Mag..

[72] Samarghandian S., Afshari J.T., Davoodi S. (2011). Honey induces apoptosis in renal cell carcinoma. Pharmacogn. Mag..

[73] Tomasin R., Gomes-Marcondes M.C.C. (2011). Oral administration of Aloe vera and honey reduces Walker tumour growth by decreasing cell proliferation and increasing apoptosis in tumour tissue. Phyther Res..

[74] Jaganathan S.K., Mandal M. (2010). Involvement of non-protein thiols, mitochondrial dysfunction, reactive oxygen species and p53 in honey-induced apoptosis. Investig. New Drugs.

[75] Sadeghi-Aliabadi H., Hamzeh J., Mirian M. (2015). Investigation of Astragalus honey and propolis extract’s cytotoxic effect on two human cancer cell lines and their oncogen and proapoptotic gene expression profiles. Adv. Biomed. Res..

[76] Zhang Q., Ma S., Liu B., Liu J., Zhu R., Li M. (2016). Chrysin induces cell apoptosis via activation of the p53/Bcl-2/caspase-9 pathway in hepatocellular carcinoma cells. Exp. Ther. Med..

[77] Li X., Wang J.-N., Huang J.-M., Xiong X.-K., Chen M.-F., Ong C.-N., Shen H.M., Yang X.F. (2011). Chrysin promotes tumor necrosis factor (TNF)-related apoptosis-inducing ligand (TRAIL) induced apoptosis in human cancer cell lines. Toxicol. Vitr..

[78] Samarghandian S., Afshari J.T., Davoodi S. (2011). Chrysin reduces proliferation and induces apoptosis in the human prostate cancer cell line pc-3. Clinics (São Paulo).

[79] Shao J., Zhang A., Qin W., Zheng L., Zhu Y., Chen X. (2012). AMP-activated protein kinase (AMPK) activation is involved in chrysin-induced growth inhibition and apoptosis in cultured A549 lung cancer cells. Biochem. Biophys. Res. Commun..

[80] Huang C., Wei Y.-X., Shen M.-C., Tu Y.-H., Wang C.-C., Huang H.-C. (2016). Chrysin, Abundant in Morinda citrifolia Fruit Water-EtOAc Extracts, Combined with Apigenin Synergistically Induced Apoptosis and Inhibited Migration in Human Breast and Liver Cancer Cells. J. Agric. Food Chem..

[81] Woo K.J., Jeong Y.-J., Park J.-W., Kwon T.K. (2004). Chrysin-induced apoptosis is mediated through caspase activation and Akt inactivation in U937 leukemia cells. Biochem. Biophys. Res. Commun..

[82] Su Q., Peng M., Zhang Y., Xu W., Darko K.O., Tao T., Huang Y., Tao X., Yang X. (2016). Quercetin induces bladder cancer cells apoptosis by activation of AMPK signaling pathway. Am. J. Cancer Res..

[83] Luo C., Liu Y., Wang P., Song C., Wang K., Dai L., Zhang J.Y., Ye H. (2016). The effect of quercetin nanoparticle on cervical cancer progression by inducing apoptosis, autophagy and anti-proliferation via JAK2 suppression. Biomed. Pharmacother..

[84] Yi L., Zongyuan Y., Cheng G., Lingyun Z., Guilian Y., Wei G. (2014). Quercetin enhances apoptotic effect of tumor necrosis factor-related apoptosis-inducing ligand (TRAIL) in ovarian cancer cells through reactive oxygen species (ROS) mediated CCAAT enhancer-binding protein homologous protein (CHOP)-death receptor 5 pathway. Cancer Sci..

[85] Ranganathan S., Halagowder D., Sivasithambaram N.D. (2015). Quercetin Suppresses Twist to Induce Apoptosis in MCF-7 Breast Cancer Cells. PLoS ONE.

[86] Mishra S., Vinayak M. (2014). Ellagic acid induces novel and atypical PKC isoforms and promotes caspase-3 dependent apoptosis by blocking energy metabolism. Nutr. Cancer.

[87] Ho C.-C., Huang A.-C., Yu C.-S., Lien J.-C., Wu S.-H., Huang Y.-P., Huang H.Y., Kuo J.H., Liao W.Y., Yang J.S. (2014). Ellagic acid induces apoptosis in TSGH8301 human bladder cancer cells through the endoplasmic reticulum stress- and mitochondria-dependent signaling pathways. Environ. Toxicol..

[88] Umesalma S., Nagendraprabhu P., Sudhandiran G. (2015). Ellagic acid inhibits proliferation and induced apoptosis via the Akt signaling pathway in HCT-15 colon adenocarcinoma cells. Mol. Cell. Biochem..

[89] Dang Q., Song W., Xu D., Ma Y., Li F., Zeng J., Zhu G., Wang X., Chang L.S., He D., Li L. (2015). Kaempferol suppresses bladder cancer tumor growth by inhibiting cell proliferation and inducing apoptosis. Mol. Carcinog..

[90] Xie F., Su M., Qiu W., Zhang M., Guo Z., Su B., Liu J., Li X., Zhou L. (2013). Kaempferol promotes apoptosis in human bladder cancer cells by inducing the tumor suppressor, PTEN. Int. J. Mol. Sci..

[91] Lee H.S., Cho H.J., Yu R., Lee K.W., Chun H.S., Park J.H.Y. (2014). Mechanisms underlying apoptosis-inducing effects of Kaempferol in HT-29 human colon cancer cells. Int. J. Mol. Sci..

[92] Tu L.-Y., Bai H.-H., Cai J.-Y., Deng S.-P. (2016). The mechanism of kaempferol induced apoptosis and inhibited proliferation in human cervical cancer SiHa cell: From macro to nano. Scanning.

[93] Luo H., Rankin G.O., Li Z., Depriest L., Chen Y.C. (2011). Kaempferol induces apoptosis in ovarian cancer cells through activating p53 in the intrinsic pathway. Food Chem..

[94] Kang G.-Y., Lee E.-R., Kim J.-H., Jung J.W., Lim J., Kim S.K., Cho S.G., Kim K.P. (2009). Downregulation of PLK-1 expression in kaempferol-induced apoptosis of MCF-7 cells. Eur. J. Pharmacol..

[95] Mantovani A. (2009). Cancer: Inflaming metastasis. Nature.

[96] Friis S., Riis A.H., Erichsen R., Baron J.A., Sørensen H.T. (2015). Low-Dose Aspirin or Nonsteroidal Anti-inflammatory Drug Use and Colorectal Cancer Risk: A Population-Based, Case-Control Study. Ann. Intern. Med..

[97] Jacobs E.J., Rodriguez C., Mondul A.M., Connell C.J., Henley S.J., Calle E.E., Thun M.J. (2005). A large cohort study of aspirin and other nonsteroidal anti-inflammatory drugs and prostate cancer incidence. J. Natl. Cancer Inst..

[98] Baandrup L., Faber M.T., Christensen J., Jensen A., Andersen K.K., Friis S., Kjaer S.K. (2013). Nonsteroidal anti-inflammatory drugs and risk of ovarian cancer: Systematic review and meta-analysis of observational studies. Acta Obstet. Gynecol. Scand..

[99] Fan Y., Mao R., Yang J. (2013). NF-κB and STAT3 signaling pathways collaboratively link inflammation to cancer. Protein Cell.

[100] Saitoh Y., Martínez Bruyn V.J., Uota S., Hasegawa A., Yamamoto N., Imoto I., Inazawa J., Yamaoka S. (2010). Overexpression of NF-κB inducing kinase underlies constitutive NF-κB activation in lung cancer cells. Lung Cancer.

[101] Compagno M., Lim W.K., Grunn A., Nandula S.V., Brahmachary M., Shen Q., Bertoni F., Ponzoni M., Scandurra M., Califano A. (2009). Mutations of multiple genes cause deregulation of NF-kappaB in diffuse large B-cell lymphoma. Nature.

[102] Kendellen M.F., Bradford J.W., Lawrence C.L., Clark K.S., Baldwin A.S. (2014). Canonical and non-canonical NF-κB signaling promotes breast cancer tumor-initiating cells. Oncogene.

[103] Zha W.J., Qian Y., Shen Y., Du Q., Chen F.F., Wu Z.Z., Li X., Huang M. (2013). Galangin Abrogates Ovalbumin-Induced Airway Inflammation via Negative Regulation of NF-kappa, B. Evid.-Based Complement. Altern. Med..

[104] Khan M.S., Devaraj H., Devaraj N. (2011). Chrysin abrogates early hepatocarcinogenesis and induces apoptosis in *N*-nitrosodiethylamine-induced preneoplastic nodules in rats. Toxicol. Appl. Pharmacol..

[105] Natarajan K., Singh S., Burke T.R., Grunberger D., Aggarwal B.B. (1996). Caffeic acid phenethyl ester is a potent and specific inhibitor of activation of nuclear transcription factor NF-kappa, B. Proc. Natl. Acad. Sci. USA.

[106] Batumalaie K., Zaman Safi S., Mohd Yusof K., Shah Ismail I., Devi Sekaran S., Qvist R. (2013). Effect of gelam honey on the oxidative stress-induced signaling pathways in pancreatic hamster cells. Int. J. Endocrinol..

[107] Safi S.Z., Batumalaie K., Qvist R., Mohd Yusof K., Ismail I.S. (2016). Gelam honey attenuates the oxidative stress-induced inflammatory pathways in pancreatic hamster cells. Evid.-Based Complement. Altern. Med..

[108] Candiracci M., Piatti E., Dominguez-Barragán M., García-Antrás D., Morgado B., Ruano D., Gutiérrez J.F., Parrado J., Castaño A. (2012). Anti-inflammatory activity of a honey flavonoid extract on lipopolysaccharide-activated N13 microglial cells. J. Agric. Food Chem..

[109] Al-Waili N.S. (2004). Natural honey lowers plasma glucose, C-reactive protein, homocysteine, and blood lipids in healthy, diabetic, and hyperlipidemic subjects: Comparison with dextrose and sucrose. J. Med. Food.

[110] Al-Waili N.S., Boni N.S. (2003). Natural honey lowers plasma prostaglandin concentrations in normal individuals. J. Med. Food..

[111] Morteau O. (2000). Prostaglandins and inflammation: The cyclooxygenase controversy. Arch. Immunol. Ther. Exp. (Warsz).

[112] Kassim M., Achoui M., Mansor M., Yusoff K.M. (2010). The inhibitory effects of Gelam honey and its extracts on nitric oxide and prostaglandin E(2) in inflammatory tissues. Fitoterapia.

[113] Michaluart P., Masferrer J.L., Carothers A.M., Subbaramaiah K., Zweifel B.S., Koboldt C., Mestre J.R., Grunberger D., Sacks P.G., Tanabe T. (1999). Inhibitory effects of caffeic acid phenethyl ester on the activity and expression of cyclooxygenase-2 in human oral epithelial cells and in a rat model of inflammation. Cancer Res..

[114] García-Mediavilla V., Crespo I., Collado P.S., Esteller A., Sánchez-Campos S., Tuñón M.J., González-Gallego J. (2007). The anti-inflammatory flavones quercetin and kaempferol cause inhibition of inducible nitric oxide synthase, cyclooxygenase-2 and reactive C-protein, and down-regulation of the nuclear factor kappaB pathway in Chang Liver cells. Eur. J. Pharmacol..

[115] Xie K. (2001). Interleukin-8 and human cancer biology. Cytokine Growth Factor Rev..

[116] Liu J.-R., Ye Y.-L., Lin T.-Y., Wang Y.-W., Peng C.-C. (2013). Effect of floral sources on the antioxidant, antimicrobial, and anti-inflammatory activities of honeys in Taiwan. Food Chem..

[117] Attia W.Y., Gabry M.S., El-Shaikh K.A., Othman G.A. (2008). The anti-tumor effect of bee honey in Ehrlich ascite tumor model of mice is coincided with stimulation of the immune cells. Egypt. J. Immunol..

[118] Al-Waili N.S. (2003). Effects of daily consumption of honey solution on hematological indices and blood levels of minerals and enzymes in normal individuals. J. Med. Food.

[119] Fukuda M., Kobayashi K., Hirono Y., Miyagawa M., Ishida T., Ejiogu E.C., Sawai M., Pinkerton K.E., Takeuchi M. (2011). Jungle honey enhances immune function and antitumor activity. Evid.-Based Complement. Altern. Med..

[120] Tonks A., Cooper R., Jones K., Parton J., Tonks A. (2003). Honey stimulates inflammatory cytokine production from monocytes. Cytokine.

[121] Gannabathula S., Skinner M.A., Rosendale D., Greenwood J.M., Mutukumira A.N., Steinhorn G., Stephens J., Krissansen G.W., Schlothauer R.C. (2012). Arabinogalactan proteins contribute to the immunostimulatory properties of New Zealand honeys. Immunopharmacol. Immunotoxicol..

[122] Dvorak H.F. (1986). Tumors: Wounds that do not heal. Similarities between tumor stroma generation and wound healing. N. Engl. J. Med..

[123] Gupta M.K., Qin R.-Y. (2003). Mechanism and its regulation of tumor-induced angiogenesis. World J. Gastroenterol..

[124] Munshi R., Bhalerao S., Kalekar S., Patil T. (2014). Exploration of the angiogenic potential of honey. Br. J. Pharmacol. Res..

[125] Seno H., Oshima M., Ishikawa T., Oshima H., Takaku K., Chiba T., Narumiya S., Taketo M.M. (2002). Cyclooxygenase 2- and Prostaglandin E_2_ Receptor EP_2_-dependent Angiogenesis in Apc{Delta}716 Mouse Intestinal Polyps. Cancer Res..

[126] Eteraf-Oskouei T., Najafi M., Gharehbagheri A. (2014). Natural honey: A new and potent anti-angiogenic agent in the air-pouch model of inflammation. Drug Res. (Stuttg).

[127] Kadir E.A., Sulaiman S.A., Yahya N.K., Othman N.H. (2013). Inhibitory effects of Tualang Honey on experimental breast cancer in rats: A preliminary study. Asian Pac. J. Cancer Prev..

[128] Lu P., Weaver V.M., Werb Z. (2012). The extracellular matrix: A dynamic niche in cancer progression. J. Cell Biol..

[129] Aziz A., Rady H., Amer M., Kiwan H. (2009). Effect of Some Honey Bee Extracts on the Proliferation, Proteolytic and Gelatinolytic Activities of the Hepatocellular Carcinoma HepG2 Cell Line. Aust. J. Basic Appl. Sci..

[130] Dornelas C.A., Fechine-Jamacaru F.V., Albuquerque I.L., Magalhães H.I.F., Dias T.A., Faria M.H.G., Alves M.K., Rabenhorst S.H., de Almeida P.R., Lemos T.L. (2012). Angiogenesis inhibition by green propolis and the angiogenic effect of L-lysine on bladder cancer in rats. Acta Cir. Bras..

[131] Pedersen T.X., Leethanakul C., Patel V., Mitola D., Lund L.R., Danø K., Johnsen M., Gutkind J.S., Bugge T.H. (2003). Laser capture microdissection-based in vivo genomic profiling of wound keratinocytes identifies similarities and differences to squamous cell carcinoma. Oncogene.

[132] Martin T.A., Ye L., Sanders A.J., Lane J., Jiang W.G. (2000). Cancer Invasion and Metastasis: Molecular and Cellular Perspective. Madam Curie Biosciences Database.

[133] Price J.T., Thompson E.W. (2002). Mechanisms of tumour invasion and metastasis: Emerging targets for therapy. Expert Opin. Ther. Targets.

[134] Oršolić N., Bašić I. (2004). Antimetastatic effect of honey. Mellifera.

[135] Stetler-Stevenson W.G. (2001). The role of matrix metalloproteinases in tumor invasion, metastasis, and angiogenesis. Surg. Oncol. Clin. N. Am..

[136] Deryugina E.I., Quigley J.P. (2006). Matrix metalloproteinases and tumor metastasis. Cancer Metastasis Rev..

[137] Halbersztadt A., Haloń A., Pajak J., Robaczyński J., Rabczynski J., St Gabryś M. (2006). The role of matrix metalloproteinases in tumor invasion and metastasis. Ginekol. Polska.

[138] Moskwa J., Borawska M.H., Markiewicz-Zukowska R., Puscion-Jakubik A., Naliwajko S.K., Socha K., Soroczynska J. (2014). Polish natural bee honeys are anti-proliferative and anti-metastatic agents in human glioblastoma multiforme U87MG cell line. PLoS ONE.

[139] Santos B.L., Oliveira M.N., Coelho P.L.C., Pitanga B.P.S., da Silva A.B., Adelita T., Silva V.D., Costa Mde F., El-Bachá R.S., Tardy M. (2015). Flavonoids suppress human glioblastoma cell growth by inhibiting cell metabolism, migration, and by regulating extracellular matrix proteins and metalloproteinases expression. Chem. Biol. Interact..

[140] Xia Y., Lian S., Khoi P.N., Yoon H.J., Joo Y.E., Chay K.O., Kim K.K., Do Jung Y. (2015). Chrysin inhibits tumor promoter-induced MMP-9 expression by blocking AP-1 via suppression of ERK and JNK pathways in gastric cancer cells. PLoS ONE.

[141] Yang B., Huang J., Xiang T., Yin X., Luo X., Huang J., Luo F., Li H., Li H., Ren G. (2014). Chrysin inhibits metastatic potential of human triple-negative breast cancer cells by modulating matrix metalloproteinase-10, epithelial to mesenchymal transition, and PI3K/Akt signaling pathway. J. Appl. Toxicol..

[142] Spoerlein C., Mahal K., Schmidt H., Schobert R. (2013). Effects of chrysin, apigenin, genistein and their homoleptic copper(II) complexes on the growth and metastatic potential of cancer cells. J. Inorg. Biochem..

[143] Peng C.-Y., Yang H.-W., Chu Y.-H., Chang Y.-C., Hsieh M.-J., Chou M.-Y., Yeh K.T., Lin Y.M., Yang S.F., Lin C.W. (2012). Caffeic Acid phenethyl ester inhibits oral cancer cell metastasis by regulating matrix metalloproteinase-2 and the mitogen-activated protein kinase pathway. Evid.-Based Complement. Altern. Med..

[144] Dziedzic A., Kubina R., Kabała-Dzik A., Wojtyczka R.D., Morawiec T., Bułdak R.J. (2014). Caffeic acid reduces the viability and migration rate of oral carcinoma cells (SCC-25) exposed to low concentrations of ethanol. Int. J. Mol. Sci..

[145] Bouzaiene N.N., Jaziri S.K., Kovacic H., Chekir-Ghedira L., Ghedira K., Luis J. (2015). The effects of caffeic, coumaric and ferulic acids on proliferation, superoxide production, adhesion and migration of human tumor cells in vitro. Eur. J. Pharmacol..

[146] Fraser S.P., Hemsley F., Djamgoz M.B.A. (2016). Caffeic acid phenethyl ester: Inhibition of metastatic cell behaviours via voltage-gated sodium channel in human breast cancer in vitro. Int. J. Biochem. Cell Biol..

[147] Kuo Y.-Y., Jim W.-T., Su L.-C., Chung C.-J., Lin C.-Y., Huo C., Tseng J.C., Huang S.H., Lai C.J., Chen B.C. (2015). Caffeic Acid phenethyl ester is a potential therapeutic agent for oral cancer. Int. J. Mol. Sci..

[148] Lin H.-P., Lin C.-Y., Liu C.-C., Su L.-C., Huo C., Kuo Y.-Y., Tseng J.C., Hsu J.M., Chen C.K., Chuu C.P. (2013). Caffeic Acid phenethyl ester as a potential treatment for advanced prostate cancer targeting akt signaling. Int. J. Mol. Sci..

[149] Ho H.-H., Chang C.-S., Ho W.-C., Liao S.-Y., Wu C.-H., Wang C.-J. (2010). Anti-metastasis effects of gallic acid on gastric cancer cells involves inhibition of NF-κB activity and downregulation of PI3K/AKT/small GTPase signals. Food Chem. Toxicol..

[150] Ho H.-H., Chang C.-S., Ho W.-C., Liao S.-Y., Lin W.-L., Wang C.-J. (2013). Gallic acid inhibits gastric cancer cells metastasis and invasive growth via increased expression of RhoB, downregulation of AKT/small GTPase signals and inhibition of NF-κB activity. Toxicol. Appl. Pharmacol..

[151] Liao C.-L., Lai K.-C., Huang A.-C., Yang J.-S., Lin J.-J., Wu S.-H., Gibson Wood W., Lin J.G., Chung J.G. (2012). Gallic acid inhibits migration and invasion in human osteosarcoma U-2 OS cells through suppressing the matrix metalloproteinase-2/-9, protein kinase B (PKB) and PKC signaling pathways. Food Chem. Toxicol..

[152] Lu Y., Jiang F., Jiang H., Wu K., Zheng X., Cai Y., Katakowski M., Chopp M., To S.S. (2010). Gallic acid suppresses cell viability, proliferation, invasion and angiogenesis in human glioma cells. Eur. J. Pharmacol..

[153] Zhao B., Hu M. (2013). Gallic acid reduces cell viability, proliferation, invasion and angiogenesis in human cervical cancer cells. Oncol. Lett..

[154] Chen Y.-J., Lin K.-N., Jhang L.-M., Huang C.-H., Lee Y.-C., Chang L.-S. (2016). Gallic acid abolishes the EGFR/Src/Akt/Erk-mediated expression of matrix metalloproteinase-9 in MCF-7 breast cancer cells. Chem. Biol. Interact..

[155] Jin U.-H., Lee J.-Y., Kang S.-K., Kim J.-K., Park W.-H., Kim J.-G., Moon S.K., Kim C.H. (2005). A phenolic compound, 5-caffeoylquinic acid (chlorogenic acid), is a new type and strong matrix metalloproteinase-9 inhibitor: Isolation and identification from methanol extract of Euonymus alatus. Life Sci..

[156] Narayanaswamy R., Kok Wai L., Ismail I.S. (2015). In Silico Analysis of Selected Honey Constituents as Human Neutrophil Elastase (HNE) and Matrix Metalloproteinases (MMP 2 and 9) Inhibitors. Int. J. Food Prop..

[157] Jeong Y.-J., Choi Y., Shin J.-M., Cho H.-J., Kang J.-H., Park K.-K., Choe J.Y., Bae Y.S., Han S.M., Kim C.H. (2014). Melittin suppresses EGF-induced cell motility and invasion by inhibiting PI3K/Akt/mTOR signaling pathway in breast cancer cells. Food Chem. Toxicol..

[158] Othman N.H., Ahmed S., Sulaiman S.A. (2016). Inhibitory effects of Malaysian tualang honey and Australian/New Zealand Manuka honey in modulating experimental breast cancers induced by *N*-methyl-*N*-nitrosourea (mnu): A comparative study. Pathology.

[159] Orsolic N., Terzic S., Sver L., Basic I. (2005). Honey-bee products in prevention and/or therapy of murine transplantable tumours. J. Sci. Food Agric..

[160] Yaacob N.S., Ismail N.F. (2014). Comparison of cytotoxicity and genotoxicity of 4-hydroxytamoxifen in combination with Tualang honey in MCF-7 and MCF-10A cells. BMC Complement. Altern. Med..

[161] Yaacob S.N., Nengsih A., Norazmi M.N. (2013). Tualang honey promotes apoptotic cell death induced by tamoxifen in breast cancer cell lines. Evid.-Based Complement. Altern. Med..

[162] Fahmy M.A., Hassan N.H.A., El-Fiky S.A., Elalfy H.G. (2015). A mixture of honey bee products ameliorates the genotoxic side effects of cyclophosphamide. Asian Pac. J. Trop. Dis..

[163] Münstedt K., Voss B., Kullmer U., Schneider U., Hübner J. (2015). Bee pollen and honey for the alleviation of hot flushes and other menopausal symptoms in breast cancer patients. Mol. Clin. Oncol..

[164] Hamad R., Jayakumar C., Ranganathan P., Mohamed R., El-Hamamy M.M.I., Dessouki A.A., Ibrahim A., Ramesh G. (2015). Honey feeding protects kidney against cisplatin nephrotoxicity through suppression of inflammation. Clin. Exp. Pharmacol. Physiol..

[165] Bardy J., Slevin N.J., Mais K.L., Molassiotis A. (2008). A systematic review of honey uses and its potential value within oncology care. J. Clin. Nurs..

[166] Zidan J., Shetver L., Gershuny A., Abzah A., Tamam S., Stein M., Friedman E. (2006). Prevention of chemotherapy-induced neutropenia by special honey intake. Med. Oncol..

[167] Abdulrhman M.A., Hamed A.A., Mohamed S.A., Hassanen N.A.A. (2016). Effect of honey on febrile neutropenia in children with acute lymphoblastic leukemia: A randomized crossover open-labeled study. Complement. Ther. Med..

[168] Song J.J., Twumasi-Ankrah P., Salcido R. (2012). Systematic review and meta-analysis on the use of honey to protect from the effects of radiation-induced oral mucositis. Adv. Skin Wound Care.

[169] Van den Wyngaert T. (2012). Topical honey application to reduce radiation-induced oral mucositis: A therapy too sweet to ignore?. J. Evid. Based Dent. Pract..

[170] Sari Dogan F., Ozaydin V., Incealtin O., Guneysel O., Demireller M. (2015). A case of acute hepatitis following mad honey ingestion. Turk. J. Emerg. Med..

[171] Makpol S., Tengku Ahmad T.A.F., Jubri Z., Rejab N., Yusof N., Yusof Y.A.M. (2012). Gelam honey acting as a radioprotectant agent in gamma-irradiated human diploid fibroblasts. J. Med. Plants Res..

